# Catalytic Transfer Hydrogenation and Acid Reactions of Furfural and 5-(Hydroxymethyl)furfural over Hf-TUD-1 Type Catalysts

**DOI:** 10.3390/molecules26237203

**Published:** 2021-11-27

**Authors:** Margarida M. Antunes, Andreia F. Silva, Carolina D. Bernardino, Auguste Fernandes, Filipa Ribeiro, Anabela A. Valente

**Affiliations:** 1 Department of Chemistry, CICECO—Aveiro Institute of Materials, University of Aveiro, 3810-193 Aveiro, Portugal; andreiafreitas@ua.pt (A.F.S.); carolinabernardino@ua.pt (C.D.B.); 2 Centro de Química Estrutural, Instituto Superior Técnico, Universidade de Lisboa, Av. Rovisco Pais, 1049-001 Lisboa, Portugal; auguste.fernandes@tecnico.ulisboa.pt (A.F.); filipa.ribeiro@tecnico.ulisboa.pt (F.R.)

**Keywords:** heterogeneous catalysis, carbohydrate biomass, furfural, 5-(hydroxymethyl)furfural, furanic ethers, mesoporous silicates, TUD-1, hafnium

## Abstract

Heterogeneous catalysis, which has served well the petrochemical industry, may valuably contribute towards a bio-based economy by sustainably enabling selective reactions to renewable chemicals. Carbohydrate-containing matter may be obtained from various widespread sources and selectively converted to furanic platform chemicals: furfural (Fur) and 5-(hydroxymethyl)furfural (Hmf). Valuable bioproducts may be obtained from these aldehydes via catalytic transfer hydrogenation (CTH) using alcohols as H-donors under relatively moderate reaction conditions. Hafnium-containing TUD-1 type catalysts were the first of ordered mesoporous silicates explored for the conversion of Fur and Hmf via CTH/alcohol strategies. The materials promoted CTH and acid reactions leading to the furanic ethers. The bioproducts spectrum was broader for the reaction of Fur than of Hmf. A Fur reaction mechanism based on literature data was discussed and supported by kinetic modelling. The influence of the Hf loading and reaction conditions (catalyst load, type of alcohol H-donor, temperature, initial substrate concentration) on the reaction kinetics was studied. The reaction conditions were optimized to maximize the yields of 2-(alkoxymethyl)furan ethers formed from Fur; up to 63% yield was reached at 88% Fur conversion, 4 h/150 °C, using Hf-TUD-1(75), which was a stable catalyst. The Hf-TUD-1(x) catalysts promoted the selective conversion of Hmf to bis(2-alkoxymethyl)furan; e.g., 96% selectivity at 98% Hmf conversion, 3 h/170 °C for Hf-TUD-1(50).

## 1. Introduction

**Scheme 1:**
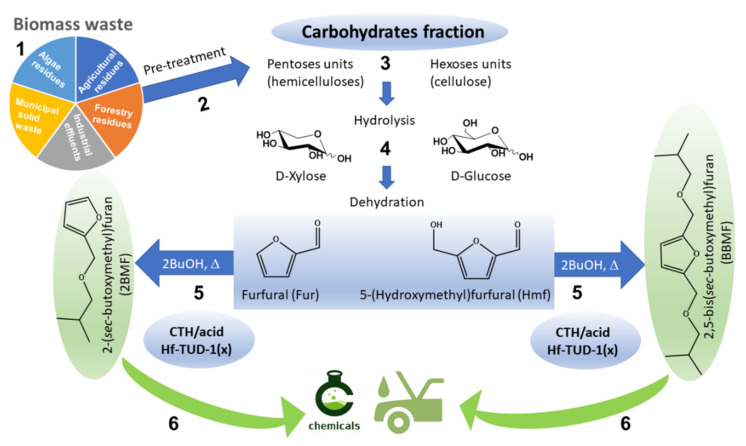
Carbohydrate biomass derived furanic aldehydes may be converted to furanic ethers (2BMF, BBMF) via catalytic transfer hydrogenation (CTH) and acid catalysis using an aliphatic alcohol as solvent and H-donor (exemplified for 2-butanol (2BuOH)) and adequate catalysts such as Hf-TUD-1(x).

The increasing greenhouse gas emissions and climate change are partly associated with the increasing energy demands and our dependency on fossil fuels [1,2]. Environmental policies have stimulated the use of renewable sources and the diversification of energy supplies. Non-edible vegetal biomass such as residues/surpluses of agriculture, forestry, and paper industries are renewable sources of organic carbon, possessing carbohydrates (CHys) such as hemicelluloses and cellulose. CHys may be selectively converted to the chemical platforms furfural (Fur) and 5-(hydroxymethyl)furfural (Hmf) via consecutive hydrolysis and dehydration reactions promoted by acid catalysts (Scheme 1) [3].

In particular, Fur has been industrially produced since 1922, and its largest volume derivative is furfuryl alcohol (FA) [4,5] which is industrially produced via copper-catalysed hydrogenation of Fur [6,7,8,9,10]. Central Romana Corporation (La Romana, Dominican Republic) has the largest Fur facility, whereas prominent Fur/FA manufacturers in Europe are International Furan Chemicals (The Hague, The Netherlands) [11] and TransFurans Chemicals (Geel, Belgium) [12,13]. FA is mainly used in the foundry industry, but it is also used for plastics, adhesives, and anti-corrosive products and as intermediate to drugs and important emerging bioproducts such as 2-(alkoxymethyl)furan ethers (AMFs) [5,7,14,15]. AMFs have promising features as fuel blends [15,16,17,18,19], flavours [20], etc.; they present a higher stability and octane number than FA [5,19,21]. Shell Oil Company claimed the zeolite-catalyzed conversion of FA to AMFs (possessing 1-4 carbon atom alkyl groups) for use as gasoline additives [21].

On the other hand, Hmf is a promising platform chemical, e.g., for replacing (toxic) formaldehyde in the production of resins for industrial use, or as intermediate for producing bioplastic alternatives to polyethylene terephthalate [22,23]. A small scale Hmf production plant was developed by AVALON Industries (Zurich, Switzerland) [24]. Somewhat in parallel to the conversion of Fur to FA, the hydrogenation of Hmf gives 2,5-bis(hydroxymethyl)furan (BHMF), which is a versatile compound for the production of resins [7,25,26], polymers [7,9,26,27,28,29], foams, fibres [9,10,26], and drugs [7,26,28,29] and for useful bioproducts such as ethers [7,19,26,27,28,30]. In particular, 2,5-bis(alkoxymethyl)furans (BAMFs) may have improved properties as diesel additives (such as higher cetane number, higher boiling point, higher stability and energy density) in relation to 5-(alkoxymethyl)furfural or ethanol [27,31,32,33]. BAMFs can be blended with commercial diesel in up to 40 vol%, contributing to reduced emissions of fumes, oxynitrides, and oxysulfides [31]. BAMFs may have more value than the respective monoethers due to the superior miscibility with diesel and higher stability of the former [32,34].

The conversion of the furanic aldehydes (Fals) to AMFs and BAMFs may be carried out via catalytic transfer hydrogenation (CTH) using an aliphatic alcohol as solvent and H-donor and using adequate catalysts (Scheme 1) [35]. These selective strategies avoid the use of expensive noble metal-containing catalysts and external supply of H_2_ which poses safety issues regarding storage, transportation, and high-pressure equipment.

Robust, thermally and chemically stable, fully inorganic ordered mesoporous silicas/silicates (OMSi) are attractive heterogeneous catalysts due to their high specific surface area, relatively narrow pore size distributions, and their mesoporous features which may favour the active sites accessibility and mass transfer and may avoid pore blockage and consequent catalyst deactivation. On the other hand, the fully inorganic characteristic of OMSi is important for Fals reaction systems which often involve the formation of insoluble humins that, at some stage, should be removed from the solid catalyst to increase the catalyst productivity. Humins may be removed from the catalysts via thermal treatment at high temperature (>350 °C), which is an issue for several families of materials such as organic–inorganic hybrids and carbon-based ones. Literature studies for the CTH of Fals focused on hexagonally arranged OMSi of the type SBA-15 [32,36,37,38,39,40,41] and MCM-41 [42,43,44] possessing zirconium and using 2-propanol (2PrOH) or 2-butanol (2BuOH) as H-donor. The preparation of these types of OMSi generally requires the use of relatively expensive copolymer organic templates or synthetic surfactant templates, leading to environmental concerns (toxic, non-biodegradable) and requiring more demanding wastewater treatments.

TUD-1 materials possess a three-dimensional sponge-like mesoporous structure and may be synthesized in a relatively inexpensive and straightforward one-pot synthesis approach without using surfactants, expensive polymeric templates, or HCl [45,46]. Several metals may be incorporated in TUD-1 to confer the desired catalytic activity. According to experimental/theoretical literature studies for different families of materials tested for CTH of Fur, hafnium-based materials may be superior catalysts than other transition metals (e.g., Zr, Ti) containing analogues [47,48,49], which may be due to factors such as enhanced binding affinity of Hf sites-reactants, compared with Zr sites [49].

Motivated by these studies, in this work, hafnium-containing OMSi type catalysts were explored for the conversion of Fur and Hmf via CTH/alcohol strategies. Due to the above-mentioned advantages, the TUD-1 family was chosen and hafnium was incorporated in different loadings (Hf-TUD-1(x), x = Si/Hf = 25, 50 or 75). The materials promoted CTH and acid reactions leading to furanic ethers. The influence of the molar ratio Si/Hf of the materials and the reaction conditions (temperature, type of alcohol H-donor, catalyst dosage, initial substrate concentration) on the reaction kinetics was studied to maximize the yields of furanic ethers. The bioproducts spectrum was broader for the reaction of Fur than of Hmf. A Fur reaction mechanism based on literature data was discussed and supported by kinetic modelling. To the best of our knowledge, this is the first study of Hf-containing OMSi catalysts used for the conversion of Fur or Hmf via CTH/alcohol strategies.

## 2. Results and Discussion

### 2.1. Catalyst Characterization

**Figure 1:**
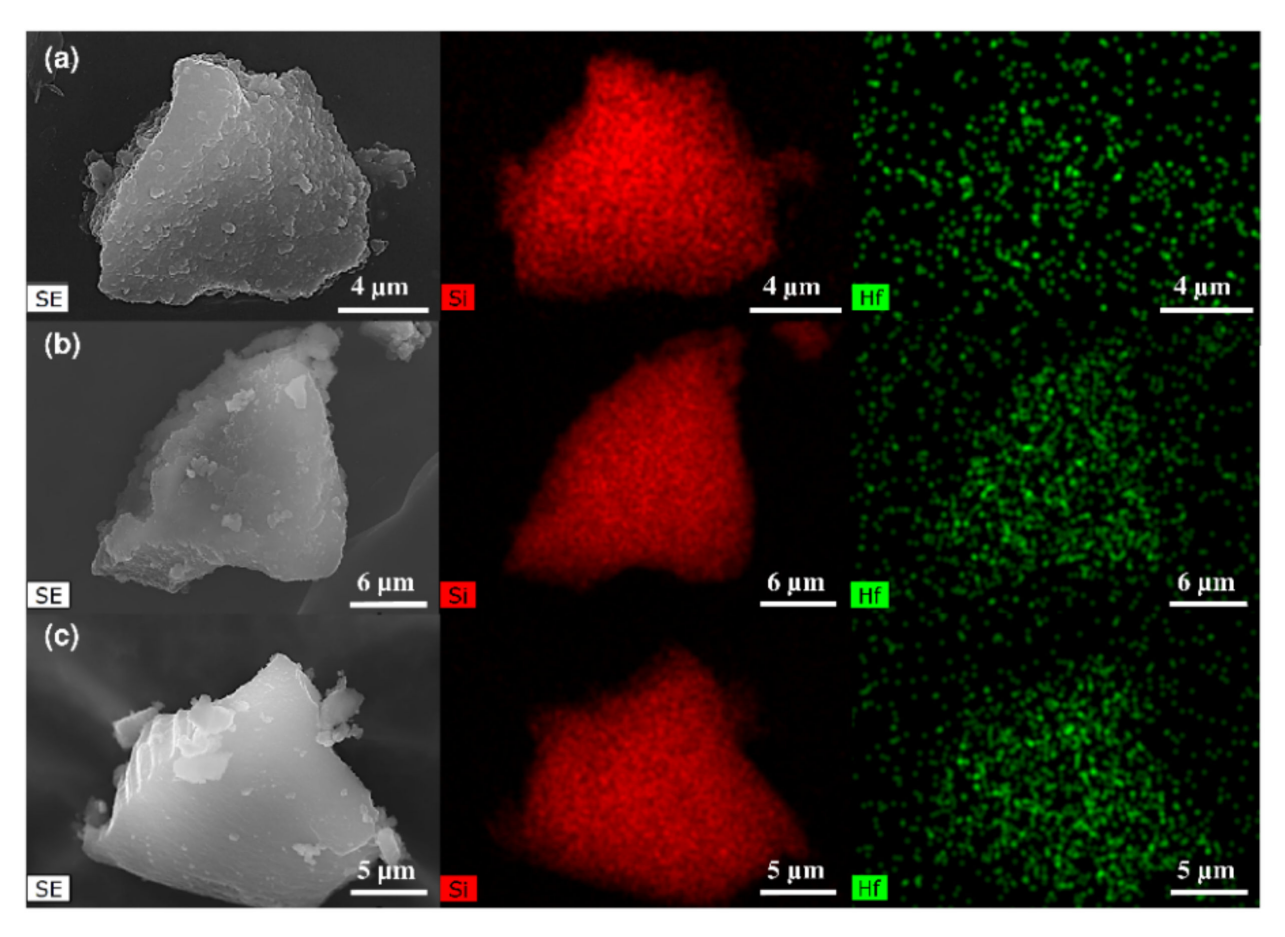
SEM images and elemental maps (Si—red, Hf—green) of Hf-TUD-1(75) (**a**), Hf-TUD-1(50) (**b**), and Hf-TUD-1(25) (**c**).

The hafnium-containing TUD-1 type materials (Hf-TUD-1(x)) were prepared by the sol–gel technique, using synthesis mixtures with molar ratios Si/Hf (x) of 25, 50, or 75; the final materials possessed Si/Hf = 67, 44, and 23, respectively (Table 1). The SEM images showed that the materials consisted of micron-size particles of irregular morphology (Figure 1 and Figure S3). A micron-scale analysis by SEM (Figure 1) and a nanoscale analysis by STEM (Figure S4) suggested that Hf-TUD-1(x) possessed uniform distributions of Si and Hf. Indeed, PXRD did not evidence the presence of HfO_2_ nanoparticles in the materials. Specifically, the wide-angle PXRD patterns showed a very broad, weak peak centred at ca. 24° 2θ, characteristic of OMSi possessing amorphous pore walls (Figure S5). On the other hand, low-angle PXRD (inset of Figure S5) showed a peak in the range 0.8–1.1° 2θ, associated with the defined mesopore size distributions of Hf-TUD-1(x) (discussed ahead).

**Figure 2:**
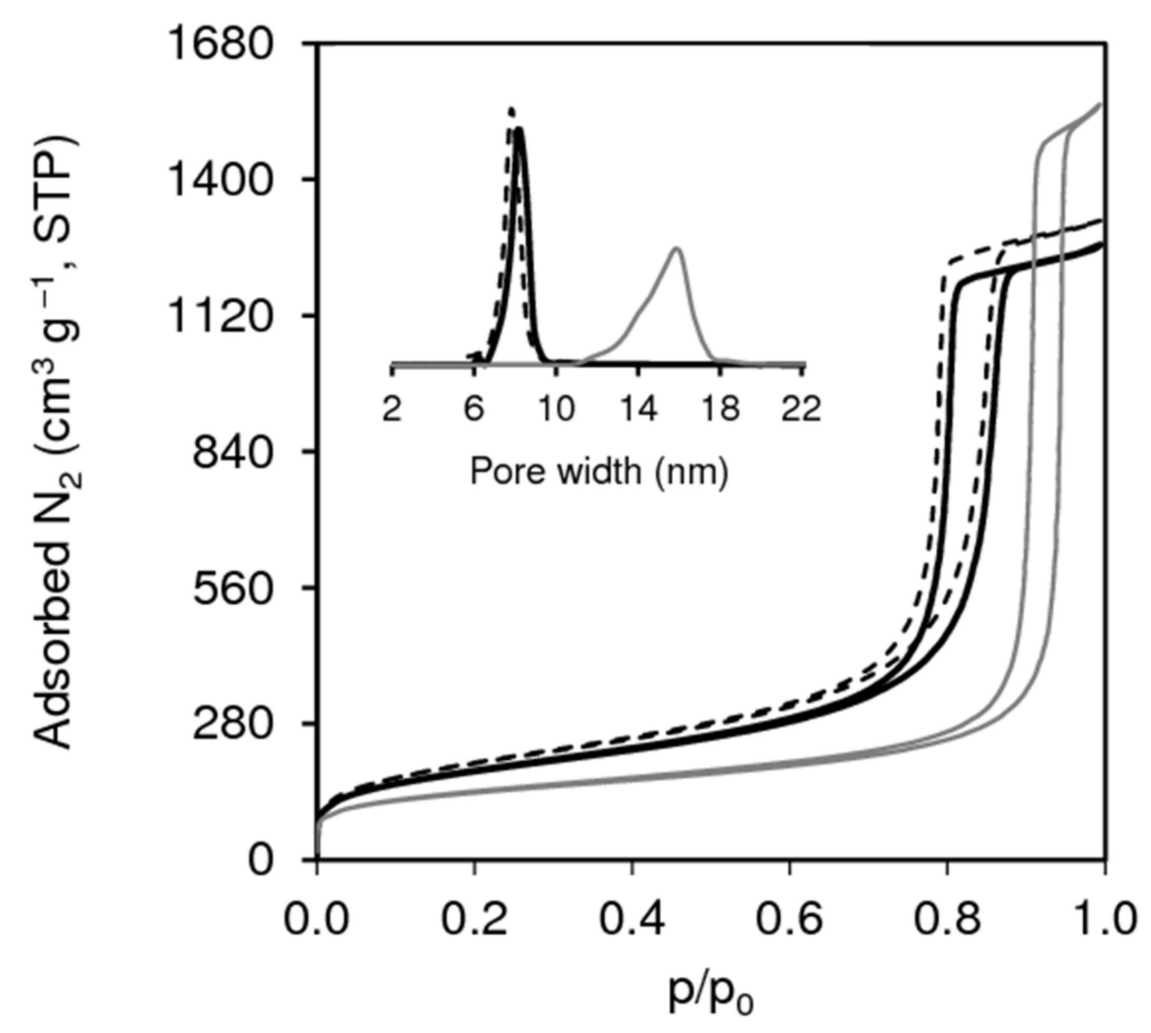
Nitrogen sorption isotherms at −196 °C of Hf-TUD-1(75) (grey solid line), Hf-TUD-1(50) (black solid line) and Hf-TUD-1(25) (black dashed line). Inset shows the corresponding mesopore size distributions (using the same lines and patterns).

The materials exhibited N_2_ sorption isotherms of type IV with a hysteresis loop, characteristic of OMSi materials (Figure 2). The median mesopore size distributions were in the range of ca. 6–10 nm for Hf-TUD-1(25) and Hf-TUD-1(50), and of ca. 11–18 nm for Hf-TUD-1(75) (inset of Figure 2). The specific surface area increased with decreasing x, specifically, from 490 m^2^ g^−1^ for x = 75 to 721 m^2^ g^−1^ for x = 25; and the pore volume was in the range 2.0–2.4 cm^3^ g^−1^, being highest for x = 75 (Table 1). These results are in agreement with literature data for TUD-1 type materials [50,51,52,53].

Figure S8 shows the FT-IR spectra of adsorbed pyridine (base probe) at 150 °C for the prepared Hf-TUD-1(x) catalysts. The materials exhibited bands at ca. 1540 and 1455 cm^−1^ due to pyridine adsorbed on Brønsted (B) and Lewis (L) acid sites, respectively [54,55]. The amount of total L plus B acid sites of Hf-TUD-1(x) increased with decreasing x (the L+B values were in the range 105–181 µmol g^−1^, Table 1). The materials possessed mostly L acidity (molar ratio L/B of 7.6–12.0) of moderate-weak strength (L_350_/L_150_ of 0.05–0.06) and did not possess strong B acid sites (B_350_/B_150_ = 0 for the three catalysts). The Lewis acidity may be essentially associated with the Hf sites of the materials, which were essential for triggering the catalytic reactions (Section 2.2.1 and Section 2.2.2).

For comparison, Zr-containing TUD-1—namely, Zr-TUD-1(50)—was prepared from a synthesis mixture with x = 50; the final material possessed Si/Zr = 55. The characterization studies indicated that Zr-TUD-1(50) was roughly comparable with Hf-TUD-1(50) in terms of structure (Figure S6a), morphology and uniform Si and Hf distributions (Figure S7), acidity (L = 113 µmol g^−1^, B = 11 µmol g^−1^; L/B = 10.3; L_350_/L_150_ = 0.10; B_350_/B_150_ = 0), and some textural properties (V_p_ = 1.9 cm^3^ g^−1^ and mesopore size range of 7–9 nm, Figure S6b). A significant difference was the higher specific surface area of Zr-TUD-1(50) in relation to Hf-TUD-1(50) (742 and 660 m^2^ g^−1^, respectively).

**Figure 3:**
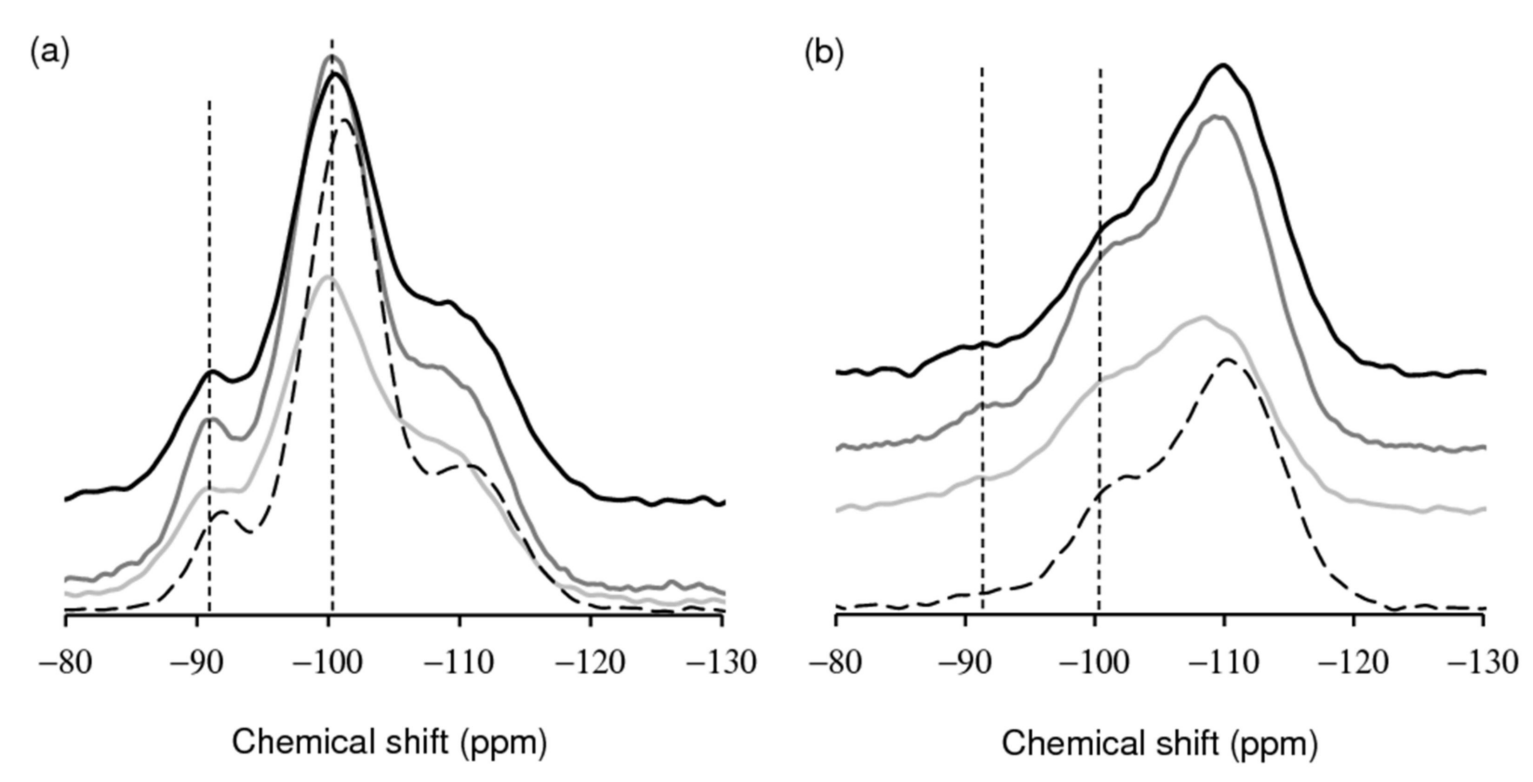
^29^Si{^1^H} CP MAS (**a**) and ^29^Si MAS NMR (**b**) spectra of Hf-TUD-1(75) (black line), Hf-TUD-1(50) (dark grey line), Hf-TUD-25 (light grey line), and TUD-1 (dashed black line).

The ^29^Si {^1^H} CP MAS NMR and ^29^Si MAS NMR spectra show peaks at approximately −110, −100, and −91 ppm, assignable to Q^n^ species (Q^n^ = Si(OSi)_n_(-OX)_4-n_) with n = 4, 3, and 2, respectively (Figure 3) [56,57,58,59]. The ^29^Si MAS NMR spectra show a prominent peak at ca. −110 ppm attributed to Q^4^ species (i.e., siloxane groups), whereas the ^29^Si {^1^H} CP MAS spectra show a prominent peak at ca. −100 ppm attributed to Q^3^ species (e.g., Si(OSi)_3_(-OH)). Some signals downfield from −100 ppm may be associated with different Q^2^ species [60]. The introduction of Hf led to downfield shifts of the signals at ca. −100 ppm and −91 ppm, in relation to siliceous TUD-1 (Figure 3a), suggesting that these signals may be related to Si sites influenced by proximal Hf atoms; e.g., ca. −91.9 ppm for siliceous TUD-1 compared with −91.2 ppm for Hf-TUD-1(50) and Hf-TUD-1(75), and 90.8 ppm for Hf-TUD-1(25).

### 2.2. Catalytic Studies

The Hf-TUD-1(x) materials were explored for the conversion of the furanic aldehydes Fur and Hmf in alcohol media. The influence of the Si/Hf molar ratio and reaction conditions (temperature, type of alcohol H-donor, catalyst dosage, initial substrate concentration) on the reaction kinetics and bioproducts distributions were investigated for the Fur reaction system. Some of the optimized Fur reaction conditions (150 °C, 20 g_cat_ L^−1^, 0.2 M substrate) were used to study the conversion of Hmf.

#### 2.2.1. Furfural Conversion

The conversion of Fur in the presence of the Hf-TUD-1(x) catalysts, using an aliphatic alcohol as solvent and H-donor in the reaction temperature range of 90–110 °C, gave the bioproducts furfuryl alcohol (FA), 2-(alkoxymethyl)furan ethers (AMFs), levulinate esters (LEs), angelica lactones (AnLs), and levulinic acid (LA), the distributions of which depended on the type of catalyst and reaction conditions used.

Whereas the Hf-TUD-1(x) effectively promoted the reaction of Fur (e.g., Hf-TUD-1(75) led to 81% conversion and a total bioproducts yield of 80% at 150 °C/3 h, discussed ahead), siliceous TUD-1 and the bulk metal oxide HfO_2_ were ineffective (<2% conversion at 4 h). Hence, the Hf sites incorporated in Hf-TUD-1(x) were essential for triggering the catalytic reaction.

**Scheme 2:**
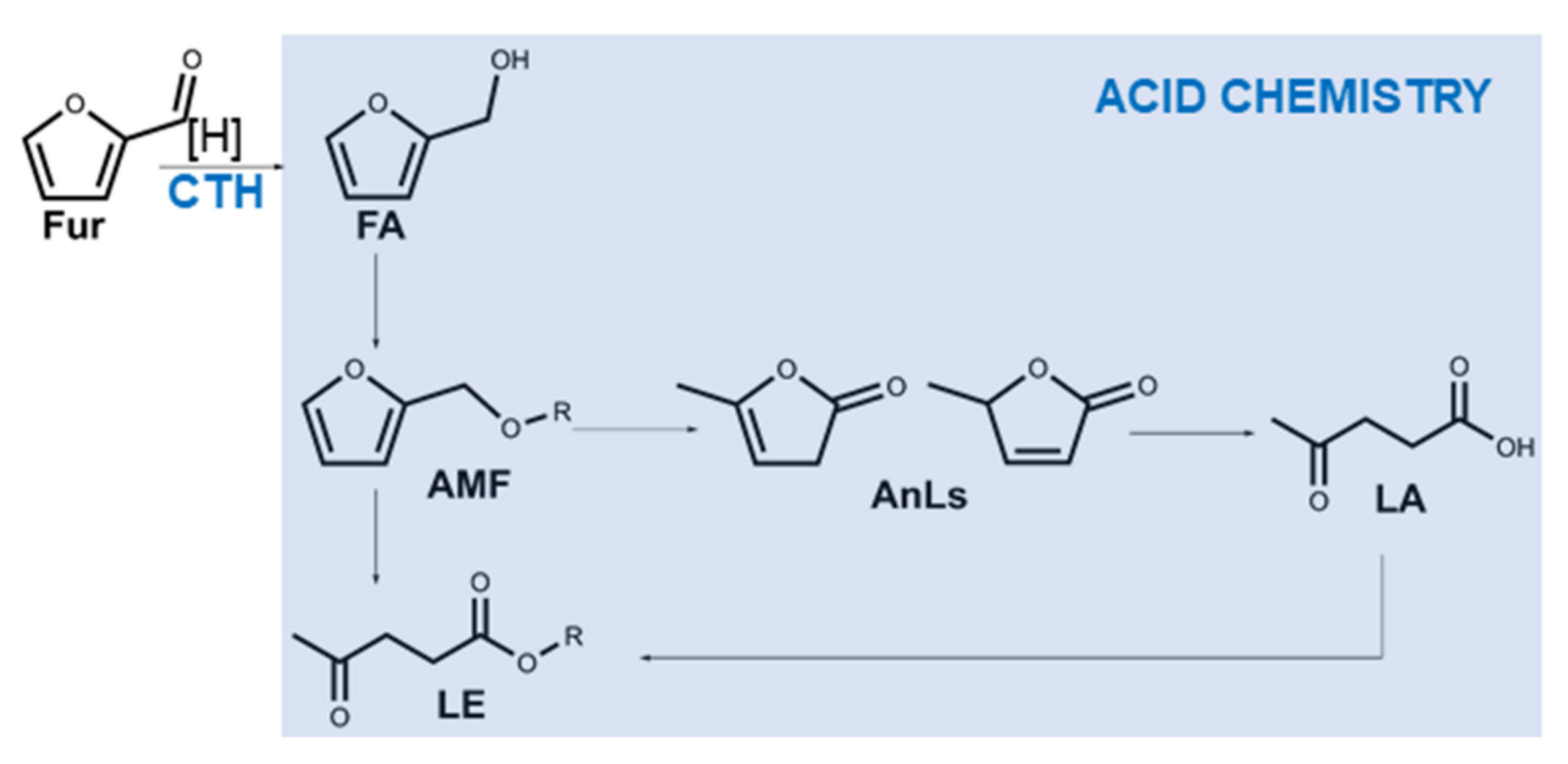
Overall reaction mechanism of Fur conversion via CTH/alcohol strategies supported by the literature and the kinetic modelling studies in this work (R = alkyl group).

According to the literature, the conversion of Fur via CTH/alcohol strategies may proceed via an overall reaction mechanism of the type presented in Scheme 2 [61,62,63,64]. This mechanism is consistent with the experimental kinetic studies discussed ahead and supported by kinetic modelling studies. Specifically, a pseudo-homogeneous kinetic model was developed (described in the Supplementary Materials), which fitted reasonably well the experimental data for Fur/2BuOH reaction at 150 °C (F_obj_ = 0.0064, Figure S2), suggesting that the mechanism in Scheme 2 is reasonable and plausible. The conversion of Fur involves CTH (Fur to FA) and acid reactions (e.g., etherification of FA to AMFs), requiring multifunctional catalysts for the one-pot synthesis of AMFs. The calculated reaction rate constants are given in Table S1. The kinetic model predicted that the fastest step of the overall process was FA to 2BMF (FA is a relatively reactive intermediate [61,62]).

##### Influence of the Reaction Temperature

**Figure 4:**
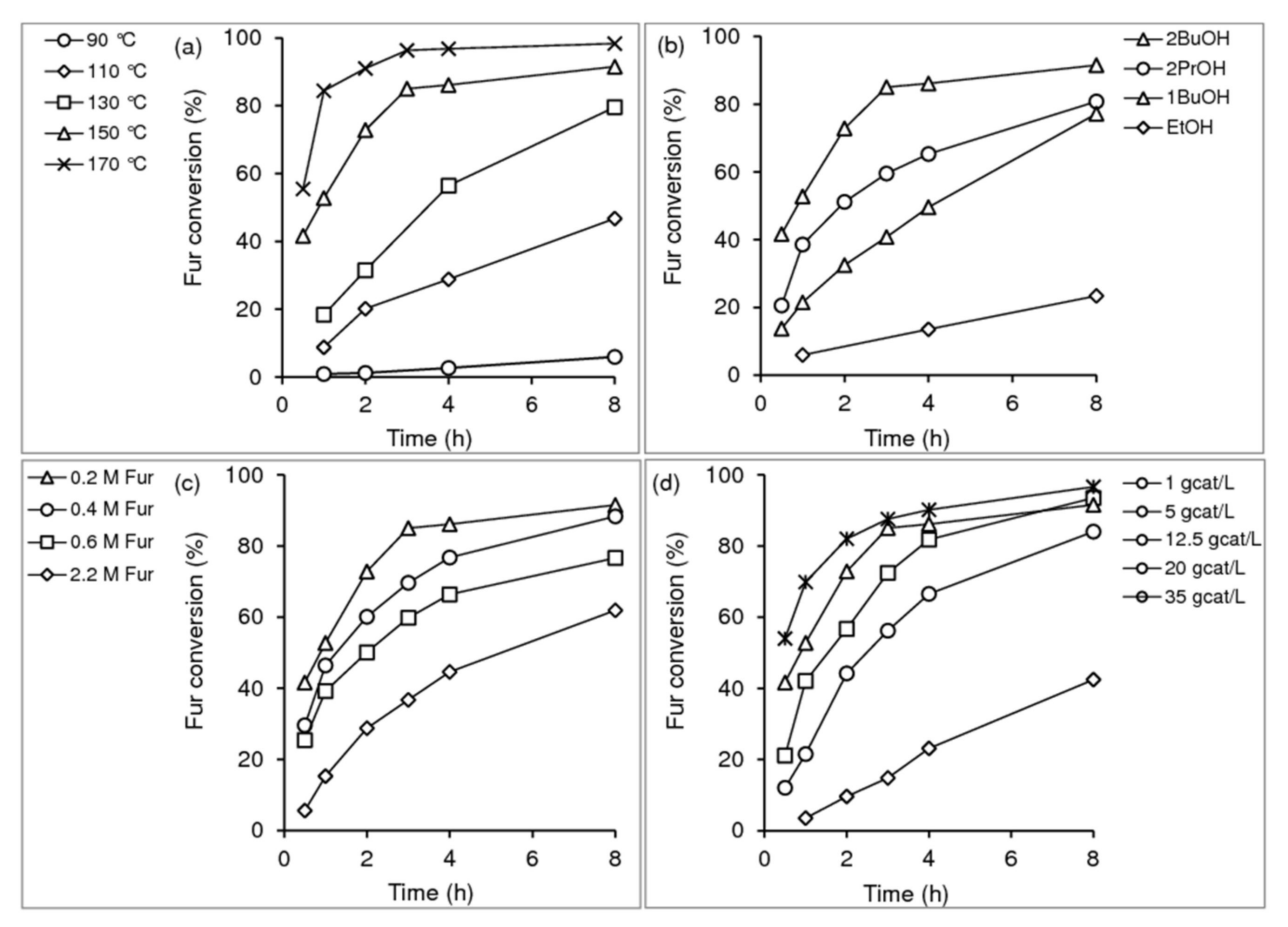
Influence of the reaction temperature (**a**), type of solvent (**b**), initial Fur concentration (**c**), and catalyst dosage (**d**) on the conversion of Fur in the presence of Hf-TUD-1(50). Reaction conditions: (**a**) 0.2 M Fur in 2BuOH, 20 g_cat_ L^−1^; (**b**) 0.2 M Fur, 20 g_cat_ L^−1^, 150 °C; (**c**) 2BuOH, 20 g_cat_ L^−1^, 150 °C; (**d**) 0.2 M Fur in 2BuOH, 150 °C. Results up to 72 h are given in the Supplementary Materials.

**Figure 5:**
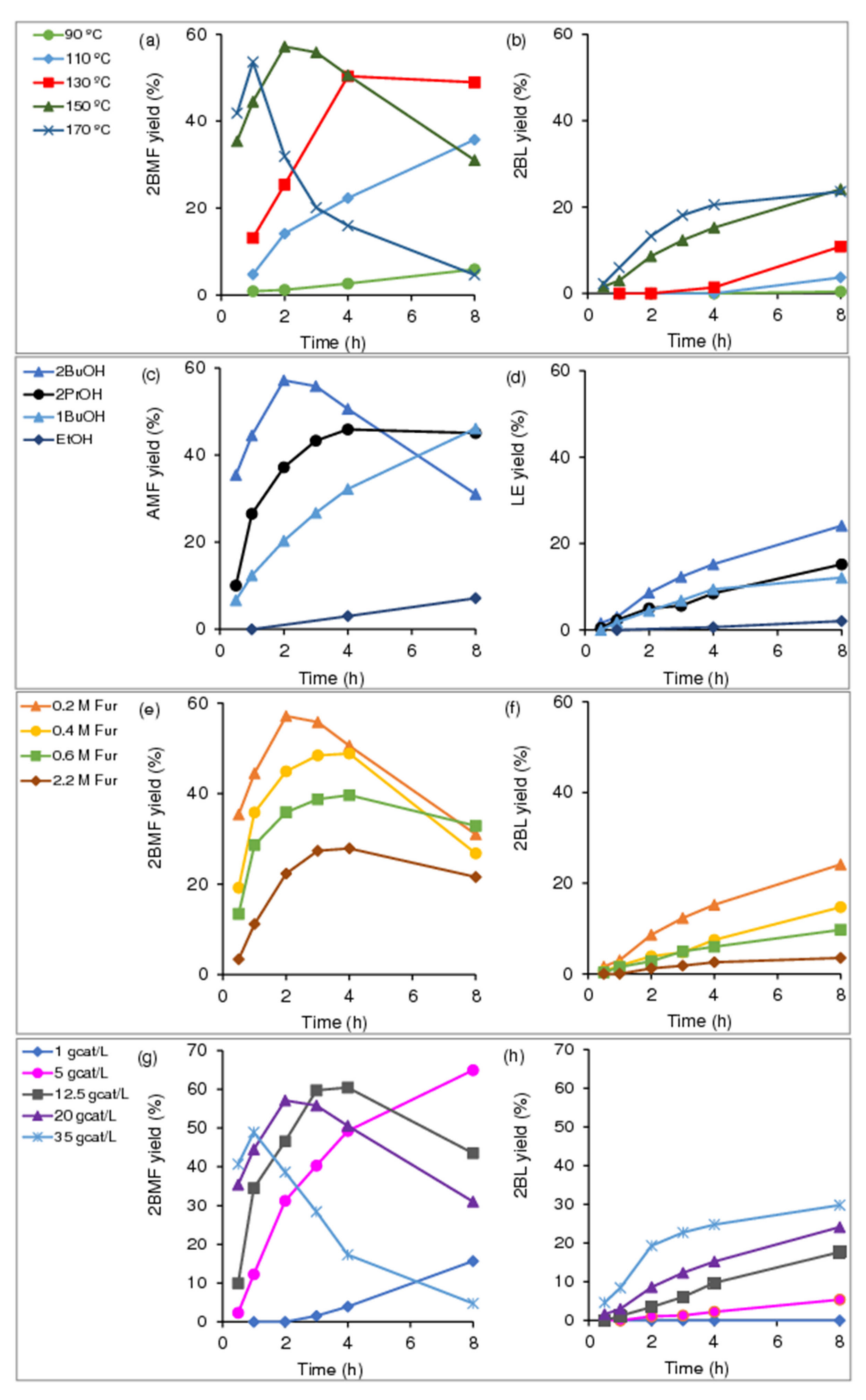
Influence of the reaction temperature (**a**,**b**), type of solvent (**c**,**d**), initial Fur concentration (**e**,**f**), and catalyst dosage (**g**,**h**) on the yields of AMFs (**a**,**c**,**e**,**g**) and LEs (**b**,**d**,**f**,**h**) for the reaction of Fur, in the presence of Hf-TUD-1(50). Reaction conditions: (**a**,**b**) 0.2 M Fur in 2BuOH, 20 g_cat_ L^−1^; (**c**,**d**) 0.2 M Fur, 20 g_cat_ L^−1^, 150 °C; (**e**,**f**) 2BuOH, 20 g_cat_ L^−1^, 150 °C; (**g**,**h**) 0.2 M Fur in 2BuOH, 150 °C. Results up to 72 h and for FA, AnLs, and LA are given in the Supplementary Materials.

The influence of the temperature on the reaction of Fur (0.2 M) was studied using Hf-TUD-1(50) as catalyst (20 g_cat_ L^−1^) and 2BuOH (Figure 4a and Figure 5a,b), which is a favourable solvent and H-donor for CTH-based strategies [61,65]. Increasing the reaction temperature in the range 90–170 °C led to increased initial activity (0.2, 1.1, 1.6, 5.4, and 7.2 mmol g_cat_^−1^ h^−1^ at 90 °C, 110 °C, 130 °C, 150 °C, and 170 °C, respectively); and Fur conversion reached 30% at 24 h/90 °C, 94% at 24 h/130 °C, 92% at 8 h/150 °C, and 91% at 2 h/170 °C (Figure 4a and Figure S9a). The kinetic results fitted reasonably well an Arrhenius plot, giving an apparent activation energy of ca. 57 kJ mol^−1^ (R^2^ = 0.994), which is in the same range of order as that reported in the literature for different types of heterogeneous catalysts tested for Fur conversion via CTH/alcohol strategies, e.g., Ni-Mg-Al catalysts for Fur to FA at 100–160 °C (batch mode, using 2PrOH) [66], and transition metal-containing Beta for Fur to FA/AMF at 55–85 °C (continuous flow, using 2PrOH) [49].

Fur was converted to FA, albeit the latter was present in low concentrations (<2% yield, Figure S10c). The main bioproduct was 2-(*sec*-butoxymethyl)furan (2BMF) formed via etherification of FA. Consistently, the kinetic model predicted that the fastest step of the overall reaction was FA to 2BMF (Table S1). The kinetic curves for 2BMF show a maximum of yield in the range 47–57% (Figure S10a). The kinetic curves of 2BL formation show an induction period for the lower reaction temperature range of 90–130 °C (Figure 5b and Figure S10b). At higher temperatures (130–170 °C), 2BMF yield decreased with the concomitant increase in 2BL yield (e.g., 31% 2BL yield was reached at 150 °C/2 h) (Figure 5a,b and Figure S10a,b). Hence, 2BMF was an intermediate leading to 2BL, consistent with Scheme 2.

The favourable formation of 2BMF may be partly due to the fact that Hf-TUD-1(50) was essentially a Lewis acid catalyst (Table 1) [43,61,67,68]. The furan ring-opening of AMFs to LEs may be favoured by B acidity [38,43,61,68,69,70], albeit Hf-TUD-1(50) possessed poor B acidity.

The kinetic profiles for the reaction at 130 and 150 °C suggested that AnL was an intermediate to LA since AnL consumption was accompanied by increasing LA yield (Figure S10d,e). These kinetic features are consistent with the mechanism presented in Scheme 2. AnL and LA were formed in low yields (10% and 15%, respectively, at 170 °C/8 h). A longer induction period was verified for LA than for 2BL, suggesting that the shorter pathway to 2BL, i.e., 2BMF-2BL, may predominate over the longer one, i.e., 2BMF-AnLs-LA-2BL (Scheme 2).

Based on the above studies, the reaction temperature of 150 °C seemed a good compromise, affording a relatively high 2BMF yield of 57% at 2 h (Figure 5a) and the carbon molar balance closed in 99%.

##### Type of Solvent/H-Donor

The influence of the type of the solvent and H-donor on the reaction of Fur (0.2 M) was studied at 150 °C (Figure 4b and Figure 5c,d). Primary alcohols (primROH) and secondary alcohols (secROH) were tested—namely, 2BuOH, 2PrOH, 1BuOH, and EtOH. The initial activity (mmol g_cat_^−1^ h^−1^) of Hf-TUD-1(50) increased in the order EtOH (0.6) < 1BuOH (2.2) < 2PrOH (3.9) < 2BuOH (5.4), indicating that the secROH were more favourable than the primROH for Fur conversion (Figure 4b and Figure S9b). A somewhat comparable trend was reported for Fur hydrogenation over the organic–inorganic hybrid catalyst Hf-H3IDCT [47]. The enhanced reaction kinetics using secROH may be partly due to the formation of more stable transition states [36], their lower reduction potential (stronger hydrogen donation capacity) in relation to primROH [71], and/or competitive adsorption effects (hydrophobicity/polarity effects associated with the carbonyl substrate versus the reductant alcohol) [72].

The type of bioproducts formed wase similar for the different solvents, but the dependency of the bioproducts distributions on reaction time changed. For secROH and 1BuOH, the main product was the AMF within 8 h (yields of up to 52–57%, Figure 5c and Figure S10f). The Fur/EtOH reaction gave the acetal 2-(diethoxymethyl)furan (up to 12% yield at 23% conversion, 8 h); and EMF and EL were formed in 22 and 25% yield, respectively, at 24 h (Figure S10f,g). For Fur/1BuOH, the acetal 2-(dibutoxymethyl)furan was formed in a negligible amount (ca. 1% at 24 h), and for Fur/secROH, acetals were not formed in measurable amounts. The formation of the acetal in the Fur/EtOH reaction was reported in the literature for Zr-SBA-15, Zr-Al-SBA-15 [70,73], and magnetic nanoparticles (Fe_3_O_4_-12) [74] at 120–160 °C. For the different solvents, AnL was formed in less than 11%, and LA was only formed using secROH (ca. 5% yield at 24 h) (Figure S10i,j).

It is desirable to avoid the decomposition of the alcohol solvent. Undesirable by-products were formed from the solvents via etherification side reactions—namely, 1,1-diethoxyethane (for EtOH), butoxybutane and dibutoxybutane (for 1BuOH), 2-*sec*-(butoxy)butane (for 2BuOH), and isopropoxypropane and diisopropoxymethane (for 2PrOH). At the maximum AMF yield, the ratio of moles of by-products/initial moles of Fur was 0.03, 0.77, 0.03, and 0.55 for 2BuOH, 2PrOH, EtOH, and 1BuOH, respectively.

Overall, using 2BuOH as solvent/H-donor led to moderate-high AMF yields at 42–86% Fur conversion, and 2BuOH presented relatively good solvent efficiency towards the target CTH reaction.

##### Influence of the Initial Concentration of Furfural

The influence of the initial concentration of Fur on the reaction kinetics was studied at 150 °C, using Hf-TUD-1(50) as catalyst (Figure 4c and Figure 5e,f). At a given reaction time, conversion decreased with increasing [Fur]_0_ in the range 0.2–2.2 M (Figure S9c). On the other hand, the initial activity increased with increasing [Fur]_0_ from 5.4 mmol g_cat_^−1^ h^−1^ for [Fur]_0_ = 0.2 M, to 19.7 mmol g_cat_^−1^ h^−1^ for [Fur]_0_ = 2.2 M (which is consistent with Equation (2) of the kinetic model; Supplementary Materials).

For increasing [Fur]_0_, the maximum yield of 2BMF decreased, and on the other hand, these maximum values were reached at progressively lower Fur conversions; specifically, from 57% maximum 2BMF yield (reached at 73% conversion) for [Fur]_0_ = 0.2 M to 28% maximum yield (reached at 45% conversion) for [Fur]_0_ = 2.2 M (Figure 5e and Figure S10k). The 2BL yield reached 31% at 100% conversion for [Fur]_0_ = 0.2 M; 2BL yields were lower for higher [Fur]_0_ (Figure S10l). LA and AnLs were always minor products (Figure S10n,o). The selectivity of total bioproducts at Fur conversion >50%, increased with decreasing [Fur]_0_. Overall, an initial Fur concentration of 0.2 M seemed favourable for targeting 2BMF.

##### Catalyst Dosage

The influence of the mass of the catalyst on the reaction of Fur (0.2 M) was studied in the range 1–35 g_cat_ L^−1^, using 2BuOH at 150 °C (Figure 4d and Figure 5g,h). The initial reaction rate (mM h^−1^) increased with increasing amount of catalyst in the order: 1 g_cat_ L^−1^ (7) < 5 g_cat_ L^−1^ (43) < 12.5 g_cat_ L^−1^ (84) < 20 g_cat_ L^−1^ (106) < 35 g_cat_ L^−1^ (140). Conversion at 8 h increased from 42% for 1 g_cat_ L^−1^ to 97% for 35 g_cat_ L^−1^ (Figure 4d). The favourable effect of a higher catalyst dosage on the reaction kinetics was reported for CTH of Fur to FA over Al-Zr@Fe mixed metal oxides [75] and the organic–inorganic hybrids Hf-PhP [76] and Hf-H3IDC-T [47].

For the lowest catalyst load of 1 g_cat_ L^−1^, FA and 2BMF were formed in 37% and 43% yield, respectively, after a very long reaction time of 72 h (Figure 5g and Figure S10p,r). Increasing catalyst dosage, i.e., a higher amount of available acids sites in the reaction medium, may favour the consecutive reactions leading to AMF and LE bioproducts [47,75]. Accordingly, increasing the catalyst dosage led to higher 2BMF yields at 1 h; 1 g_cat_ L^−1^ (0%) < 5 g_cat_ L^−1^ (12%) < 12.5 g_cat_ L^−1^ (35%) < 20 g_cat_ L^−1^ (45%) < 35 g_cat_ L^−1^ (49%). However, the maximum 2BMF yield tended to decrease with increasing catalyst dosage (excluding the very slow reaction using 1 g_cat_ L^−1^), specifically, from 65% for 5 g_cat_ L^−1^ to 49% for 35 g_cat_ L^−1^ (Figure 5g and Figure S10p). A reasonable compromise between catalyst dosage and reaction time seemed to be reached using 12.5–20 g_cat_ L^−1^, which led to 57–60% 2BMF yield within 2–3 h (ca. 73% conversion, Figure 5g); under these conditions, 2BL ((Figure 5h) and AnLs (Figure S10s) were formed with 6–9% and 4–6% yield, respectively, and LA was not formed (Figure S10t), giving a selectivity to total bioproducts of 97–99%.

##### Hafnium versus Zirconium Catalysts

The performance of Hf-TUD-1(50) was compared with that of its counterpart Zr-TUD-1(50) at 150 °C (0.2 M Fur in 2BuOH, 20 g_cat_ L^−1^). The initial activity of Zr-TUD-1(50) was greater than that of Hf-TUD-1(50) (7.3 and 5.4 mmol g_cat_^−1^ h^−1^, respectively). The two materials possessed similar structure, morphology, and acid properties (Section 2.1). Although Zr-TUD-1(50) possessed a higher specific surface area, the two catalysts possessed similar initial activity expressed per surface area (ca. 0.01 mmol m^−2^ h^−1^). However, a higher specific surface area may lead to higher adsorption capacity (i.e., amount of reactant adsorbed on the solid surface per gram of catalyst). Since the mass of catalyst was kept constant, a higher surface area was available in the reaction medium in the case of Zr-TUD-1(50), which may enhance Fur conversion in comparison with Hf-TUD-1(50).

**Figure 6:**
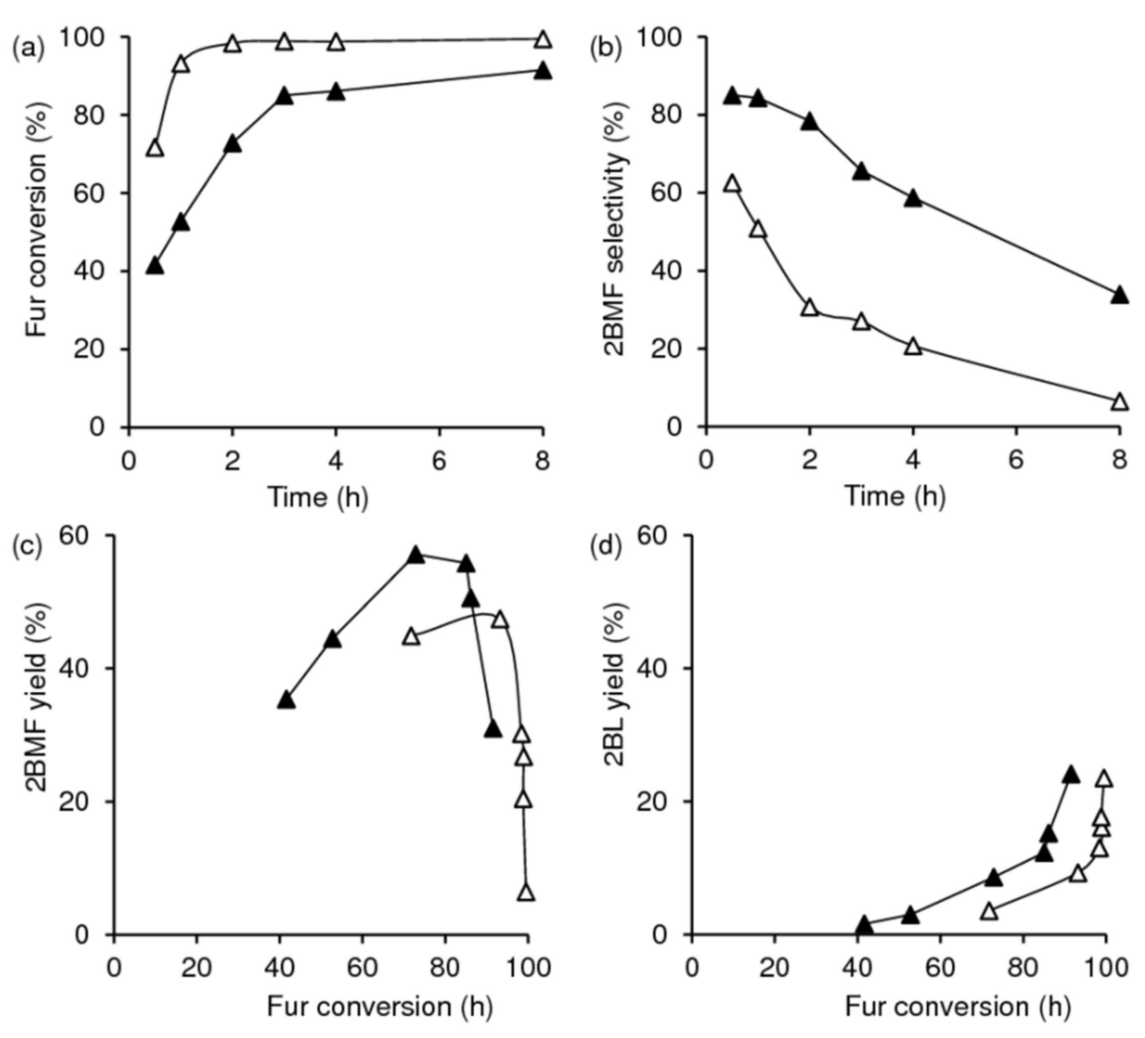
Comparison of the Hf-TUD-1(50) (▲) and Zr-TUD-1(50) (Δ) catalysts: kinetic profile of Fur (**a**); 2BMF selectivity dependency on reaction time (**b**); and the dependencies of the yields of 2BMF (**c**) and 2BL (**d**) on Fur conversion at 150 °C. Reaction conditions: 0.2 M Fur in 2BuOH, 20 g_cat_ L^−1^.

In terms of bioproducts distribution, the two catalysts led mainly to 2BMF. However, Hf-TUD-1(50) was more selective and led to a higher maximum yield of 2BMF (57% yield at 73% conversion; 78% 2BMF selectivity, 2 h) than Zr-TUD-1(50) (48% yield at 93% conversion; 52% 2BMF selectivity, 1 h) (Figure 6). At the maximum 2BMF yield, the carbon molar balance closed in a higher percentage for the Hf catalyst than for the Zr one (99% and 77%, respectively). Overall, the Hf catalyst was more attractive for targeting 2BMF.

##### Si/Hf Ratio of Hf-TUD-1 Materials

**Figure 7:**
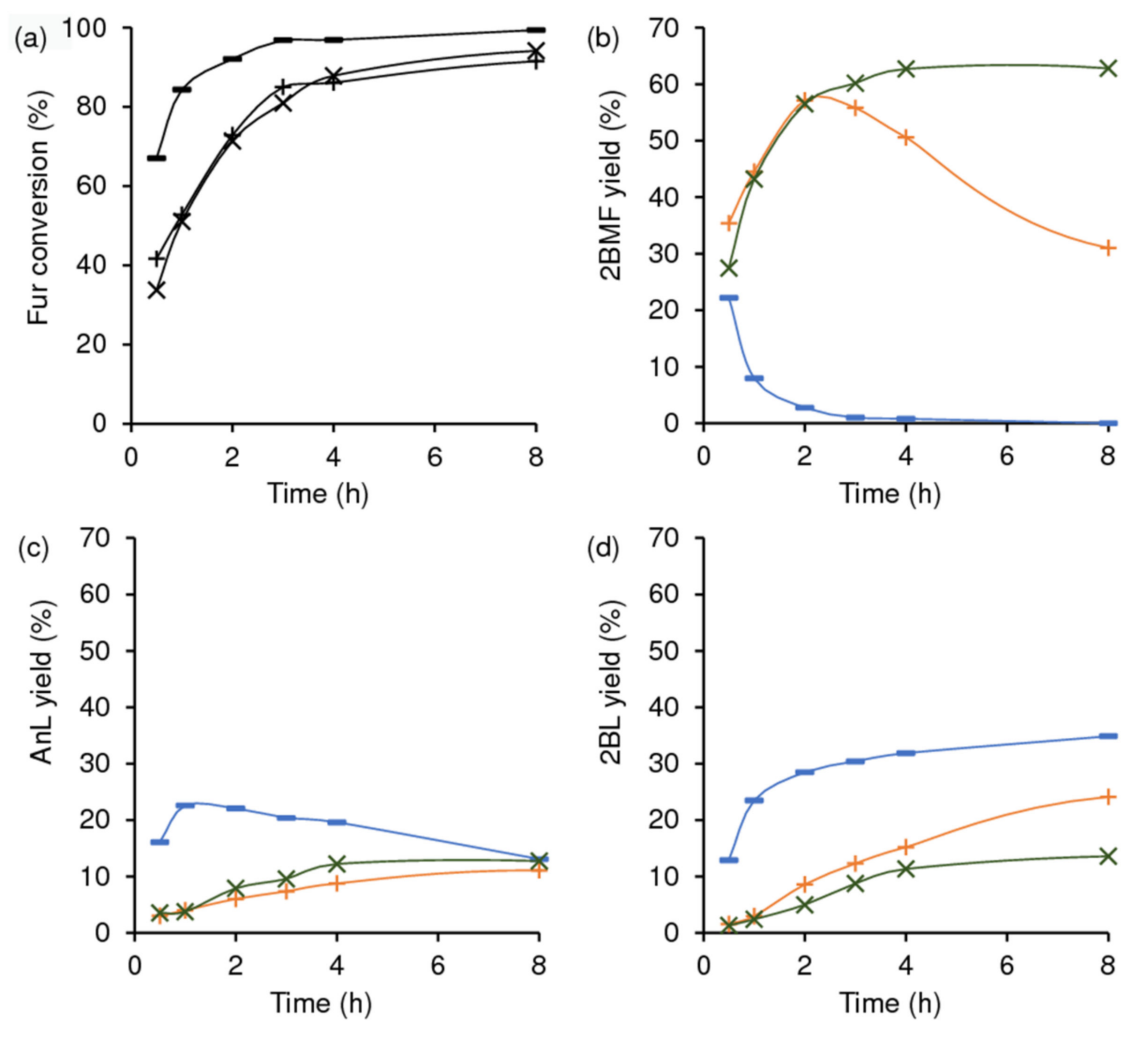
Influence of the molar ratio Si/Hf of the Hf-TUD-1(x) catalysts (x = 25 (−); x= 50 (+); x = 75 (x)) on Fur conversion (**a**) and yields of 2BMF (**b**), AnL (**c**), and 2BL (**d**) at 150 °C. Reaction conditions: 0.2 M Fur in 2BuOH, 20 g_cat_ L^−1^. Results up to 24 h and for FA and LA are given in the Supplementary Materials.

As discussed above, the Hf sites played determinant roles in the formation of the bioproducts. Hence, it is important to study the influence of the Si/Hf ratio on the catalytic performance. Hf-TUD-1(x) with x = 25 and 75 was compared with Hf-TUD-1(50) at 150 °C (0.2 M Fur in 2BuOH) (Figure 7 and Figure S11)). The initial activity decreased by increasing x from 25 to 50 (8.6 and 5.4 mmol g_cat_^−1^ h^−1^, respectively). A further increase in x from 50 to 75 did not significantly affect the initial activity (5.2 mmol g_cat_^−1^ h^−1^). For x = 25 and 50, the increasing initial activity correlated with the increasing amount of total acid sites (with decreasing x), but this correlation was not so evident for x = 50 versus 75 (Table 1). A marked difference of Hf-TUD-1(75) in relation to the remaining catalysts was its larger mesopores and greater pore volume (Table 1), which may lead to a higher volume of reactants inside the catalyst (per gram of material), favouring the overall reaction. Important improvements in the catalytic activity of OMSi by expanding the mesopore sizes were reported in the literature for Fur conversion [36].

The bioproducts spectrum was similar for the three materials. However, the selectivity to total bioproducts at 92–99% Fur conversion (8 h) increased with increasing x, from 64% for x = 25 and 77% for x = 50 to 97% for x = 75; in parallel, the percentage in which the carbon molar balance closed also increased with increasing x. The higher acidity of Hf-TUD-1(25) may favour side reactions leading to non-volatile (oligo/polymeric) by-products. On the other hand, the lower acidity and the wider mesopore sizes and greater pore volume of Hf-TUD-1(75) may avoid pore blockage by carbonaceous deposits inside the catalyst, favouring the total selectivity. Limited pore blockage was reported in the literature for a Zr-doped SBA-15 possessing enlarged mesopores (prepared with a copolymer as template and 1,3,5-trimethylbenzene as pore expander), tested for CTH of Fur to FA [36].

The formation of 2BMF was favoured with increasing x (Figure 7b and Figure S11b); in the conversion range >65%, the highest 2BMF yield reached followed the order 22% (x = 25) < 57% (x = 50) < 63% (x = 75). On the other hand, the formation of 2BL (Figure 7d and Figure S11d) and LA (Figure S11f) were favoured by decreasing x; 2BL yield at 8 h (92–99% conversion) followed the order 14% (x = 75) < 24% (x = 50) < 35% (x = 25). Moreover, Hf-TUD-1(25) led to the highest AnLs yield (23% at 84% conversion, compared with less than 13% for the remaining catalysts) (Figure 7c and Figure S11c). Hence, the most active catalyst Hf-TUD-1(25) favoured consecutive reactions of 2BMF (Scheme 2). These results somewhat parallel literature data for Zr catalysts, in that higher Si/Zr ratios led to slower reaction kinetics and lower LE yield [43,77].

In parallel to that verified for Hf-TUD-1(50), decreasing amount of Hf-TUD-1(75) favoured FA formation (Figure S12), reaching 60% FA yield at 86% conversion, 72 h, using a low catalyst dosage of 1 g_cat_ L^−1^. Lower FA yields were reached using Hf-TUD-1(50) under similar reaction conditions (37% FA yield at 86% conversion, 72 h). It seems that decreasing the catalyst dosage and increasing the Si/Hf ratio may enhance the FA yields.

The influence of the molar ratio Si/Hf on the non-productive decomposition of 2BuOH was studied. The degree of the decomposition of 2BuOH increased with decreasing x. Specifically, 2-sec-(butoxy)butane was formed in 0.02%, 0.12%, and 0.25% yield for Hf-TUD-1(75), Hf-TUD-1(50), and Hf-TUD-1(25), respectively. At the highest 2BMF yield reached for each catalyst, the ratio of moles of 2-sec-(butoxy)butane/initial moles of Fur was 0.01, 0.03, and 0.04 for x = 75, 50, and 25, respectively. Overall, Hf-TUD-1(75) was a favourable catalyst for targeting 2BMF and led to slightly lower solvent degradation.

##### Literature Survey for Fur Conversion via CHT/Alcohol Strategies

Hf-TUD-1(x) catalysts are the first Hf-mesoporous silicates reported for Fur conversion using secROH. The catalytic results for Hf-TUD-1(x) were compared with literature data for fully inorganic heterogeneous non-noble metal catalysts tested for Fur conversion to AMFs in alcohol medium (Table 2) [34,36,37,39,42,43,61,62,65,67,78,79,80,81,82,83,84,85]. The present work does not concern noble metal catalysts for Fur conversion, which may be found in a vast literature such as the following references, just to cite a few [86,87,88]. The reported heterogeneous non-noble catalysts were essentially OMSi, metal oxides, and zeolitic materials. Broad ranges of the different reaction parameters were employed (110–180 °C; different H-donors; [Fur]_0_ of 0.07–1.31 M, Cat/Fur mass ratio of 0.13–3.7). Table 2 lists the highest AMF yields reported for each study (irrespective of the type of H-donor alcohol). Hf-TUD-1(50) led to 65% AMF at 150 °C/8 h, using a Cat/Fur mass ratio of 0.25 and [Fur]_0_ of 0.2 M (entry 2); a similar 63% yield was reached by increasing x from 50 to 75 and changing the reaction conditions (entry 1).

The highest AMF yield reported in the literature was 96% for the mixed metal oxide ~70wt%ZrO_2_/SiO_2_ at 120 °C/4 h, using a Cat/Fur mass ratio of 1.0 and [Fur]_0_ of 0.13 M (entry 19 [34]). AMF yields in the range 70–89% were reported for Zr-SBA-15 (entry 6 [37]), ZrO_2_/SBA-15 (17 wt% Zr, entry 8 [39]), and MP-ZrAl-Beta-m (entry 12 [65]). The range 60–69% of AMF yields (reached using the catalysts prepared in this work) was reported for a mechanical mixture of two materials—namely, Zr-montmorillonite plus ZrO(OH)_2_ (entry 22 [82]) and a composite material Zr-AlBeta/TUD-1 (entry 25 [65]). The remaining catalysts listed in Table 2 led to lower AMF yields. Overall, the results for Hf-TUD-1(50) and Hf-TUD-1(75) as single catalysts possessing a relatively small amount of transition metal seem good, and their synthesis was performed in an integrated fashion (one-pot process) without using hazardous mineral acids and/or strong acidic conditions (as for MP-ZrAl-Beta-m, SBA-15 type materials), relatively expensive copolymeric organic templates (SBA-15), and avoiding multiple synthesis operations (MP-ZrAl-Beta-m, Zr-AlBeta/TUD-1) that require greater consumption of chemical substances and more equipment and that may generate more waste.

#### 2.2.2. 5-(Hydroxymethyl)furfural Conversion

The reaction of Hmf was studied over the Hf-TUD-1(x) catalysts at 150 °C, targeting bis(2-alkoxymethyl)furans (BAMFs). Literature studies reported the conversion of Hmf to BAMFs in alcohol medium, which may involve 2,5-bis(hydroxymethyl)furan (BHMF) as intermediate, although sometimes no measurable amounts were reported [33,34,40,41,44,82]. For the Hf-TUD-1(50) catalyst, BAMF was the only product formed in measurable amounts (up to 93% yield) under different reaction conditions (Figure S13): 2BuOH or 2PrOH; 150 or 170 °C; catalyst dosage of 20 g_cat_ L^−1^ or 1 g_cat_ L^−1^ at 150 °C(which gave 10% BAMF yield at 13% Hmf conversion, 8 h, in the presence of Hf-TUD-1(50), Figure S13d–f).

The influence of the type of solvent was studied using 2BuOH and 2PrOH, in the presence of Hf-TUD-1(50) (20 g_cat_ L^−1^) at 150 °C (Figure S13a–c). The Hmf/2BuOH reaction system led to higher conversions (Figure S13a) and BAMF yields than the Hmf/2PrOH one (Figure S13b,c). Specifically, initial activity was 3.7 and 0.8 mmol g_cat_^−1^ h^−1^, respectively, and the maximum BAMF yields reached were 79% (97% selectivity, 3 h) and 40% (43% selectivity, 24 h) using 2BuOH and 2PrOH, respectively. These results somewhat parallel those for Fur/secROH (Section 2.2.1). Increasing the Hmf/2BuOH reaction temperature from 150 to 170 °C enhanced the conversion of Hmf to bis(2-butoxymethyl)furan (BBMF) (Figure S13d); the initial activity was 3.7 and 5.1 mmol g_cat_^−1^ h^−1^, and conversion at 3 h was 80% and 98%, respectively. Higher BBMF yields were reached at 170 °C than at 150 °C; 94% and 79%, respectively, at 3 h (Figure S13e,f).

**Figure 8:**
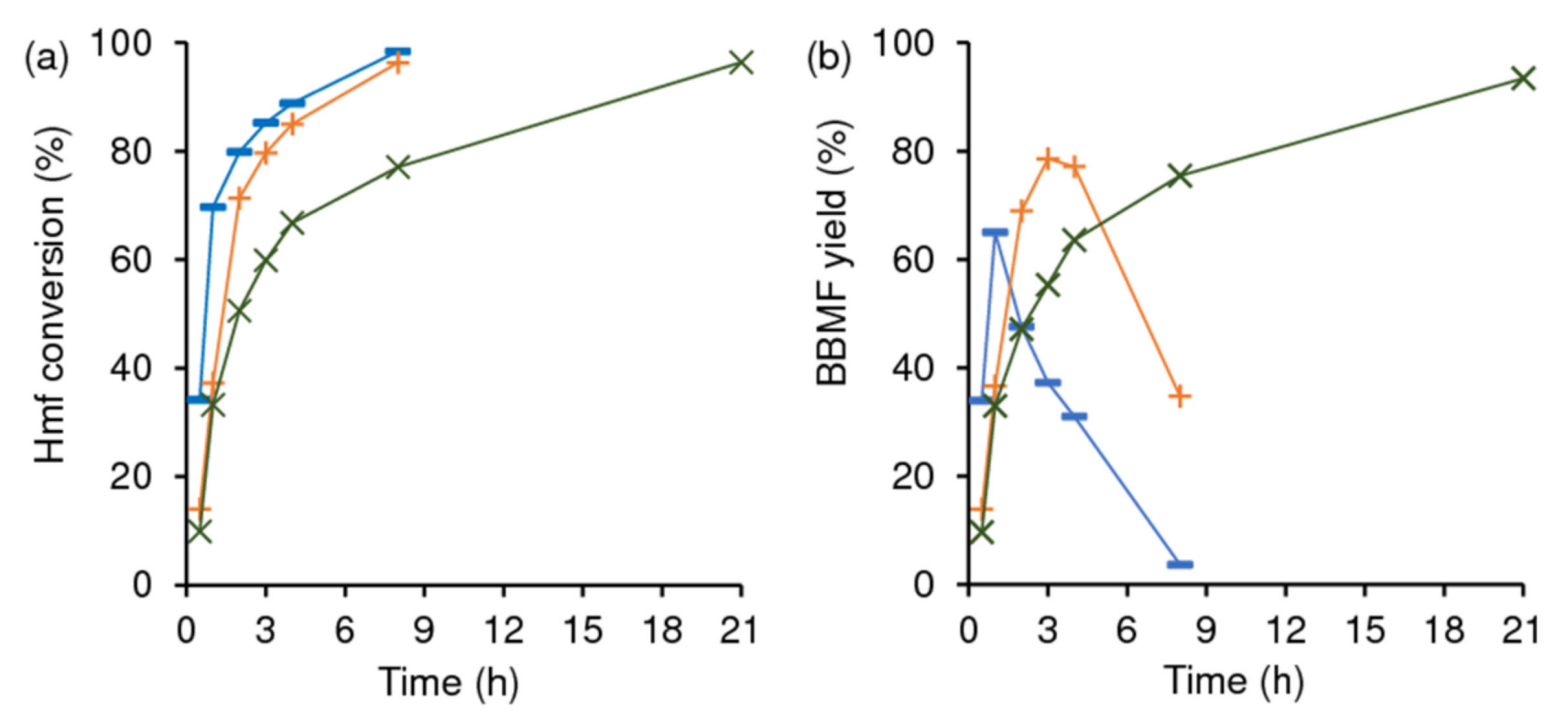
Influence of the molar ratio Si/Hf of the Hf-TUD-1(x) catalysts (x = 25 (−); x = 50 (+); x = 75 (x)) on Hmf conversion (**a**) and BBMF yield (**b**) at 150 °C. Reaction conditions: 0.2 M Hmf in 2BuOH, 20 g_cat_ L^−1^.

The influence of the Si/Hf molar ratio on the Hmf/2BuOH reaction at 150 °C was studied (Figure 8). Initial activity (mmol g_cat_^−1^ h^−1^) increased in the order Hf-TUD-1(75) (3.3) ≈ Hf-TUD-1(50) (3.7) < Hf-TUD-1(25) (7.0), somewhat in parallel to that verified for the reaction of Fur (Section 2.2.1. Increasing x led to higher maximum BBMF yields (reached at higher reaction time and conversion); from 65% yield at 70% conversion (1 h) for x = 25 and 79% yield at 80% conversion (3 h) for x = 50 to 93% yield at 96% conversion (21 h) for x = 75. On the other hand, BBMF selectivity at 96–98% conversion was 97% for x = 75 and only 36% and 5% for x = 50 and 25, respectively. Hence, Hf-TUD-1(75) was a favourable catalyst for targeting BBMF.

As with Fur as substrate, the Hf-TUD-1(x) catalysts are the first Hf-containing OMSi type materials studied for Hmf conversion via CTH/alcohol strategies. The performances of the Hf-TUD-1(x) catalysts were compared with literature data for fully inorganic non-noble heterogeneous catalysts tested for Hmf conversion to BAMFs in alcohol medium (Table 3) [32,33,34,40,41,44,82,89]. The comparisons are based on the highest BAMF yields reported for each study (irrespective of the type of alcohol H-donor). The reported catalysts were essentially OMSi and zeolitic materials.

The highest BAMF yield was reported for a Zr/Si mixed oxide catalyst (97% yield at 120 °C/7 h; entry 23, Table 3) [34]. Hf-TUD-1(75) led to a comparable yield of 93%, but at higher temperature (150 °C) and longer reaction time (21 h, entry 1). Nevertheless, an important difference between the two materials was the amount of transition metal; the reported Zr/Si mixed oxide possessed ca. 70 wt% ZrO_2_ [34], whereas Hf-TUD-1(75) possessed ca. 3.8 wt% Hf. A high BBMF yield (96%) was also reported for a solid mixture of Zr-montmorillonite plus ZrO(OH)_2_ (1:1 mass ratio) at 150 °C/1 h (entry 24) [82]. However, solid mixtures may present some difficulties in optimizing the total catalyst productivity if the lifetimes and stabilities of the solids differ. For M-zeotype catalysts, the BAMF yield did not surpass 81% (entries 12–22) [33,40,44,89].

#### 2.2.3. Catalyst Stability

The catalyst stability is very important for enhancing productivity. The Hf-TUD-1(x) catalysts used in the reaction of Fur were brownish in colour, and elemental analysis indicated that they possessed carbonaceous matter; carbon contents of 16, 9, and 6 wt.% for x = 25, 50, and 75, respectively. The presence of organic matter was also evidenced by ATR FT-IR spectroscopy. Figure S14 shows the spectra for the original, washed/dried, and washed/dried/calcined solids for a selected catalyst (namely, Hf-TUD-1(50) used in the reaction of Fur/2BuOH at 150 °C). The spectra of the original and washed/dried/calcined solids were similar, suggesting that the silicate surface chemistry was essentially preserved during the catalytic and regeneration processes. A shoulder at ca. 960 cm^−1^ is assignable to Si-(OX) vibrations with X = H or Hf [90]. The non-calcined catalyst exhibited new bands (which disappeared after calcination): ca. 1450 cm^−1^ assignable to C-H vibrations of adsorbed organic matter [90]; ca. 1740 cm^−1^ assignable to stretching vibrations of aldehyde groups [91]; and ca. 1386 cm^−1^ assignable to metal-butoxy type groups [92].

In order to remove the organic matter from the used catalysts, these were subjected to a thermal treatment (Section 3.3 for details). The Hf-TUD-1(x) catalysts recovered from the Fur/2BuOH reaction at 150 °C were characterized in a similar fashion to the respective original catalysts. The used catalysts possessed comparable structural (PXRD, Figure S15), textural and, acid (Table S2) and morphological (Figure S16) properties, metal distributions (element maps, Figure S16), and Si/Hf ratio (Table S2) to the respective original catalysts. Consistently, a contact test (using 2BuOH at 150 °C/3 h; please see Section 3.3 for details) indicated that no soluble active species were present in the liquid phase since the solution (obtained by contacting the solid catalyst with the solvent under catalytic conditions (without Fur), followed by catalyst separation and, finally, by the addition of Fur to the liquid phase without the solid catalyst) led to similar conversion to the blank test without catalyst (<1%).

**Figure 9:**
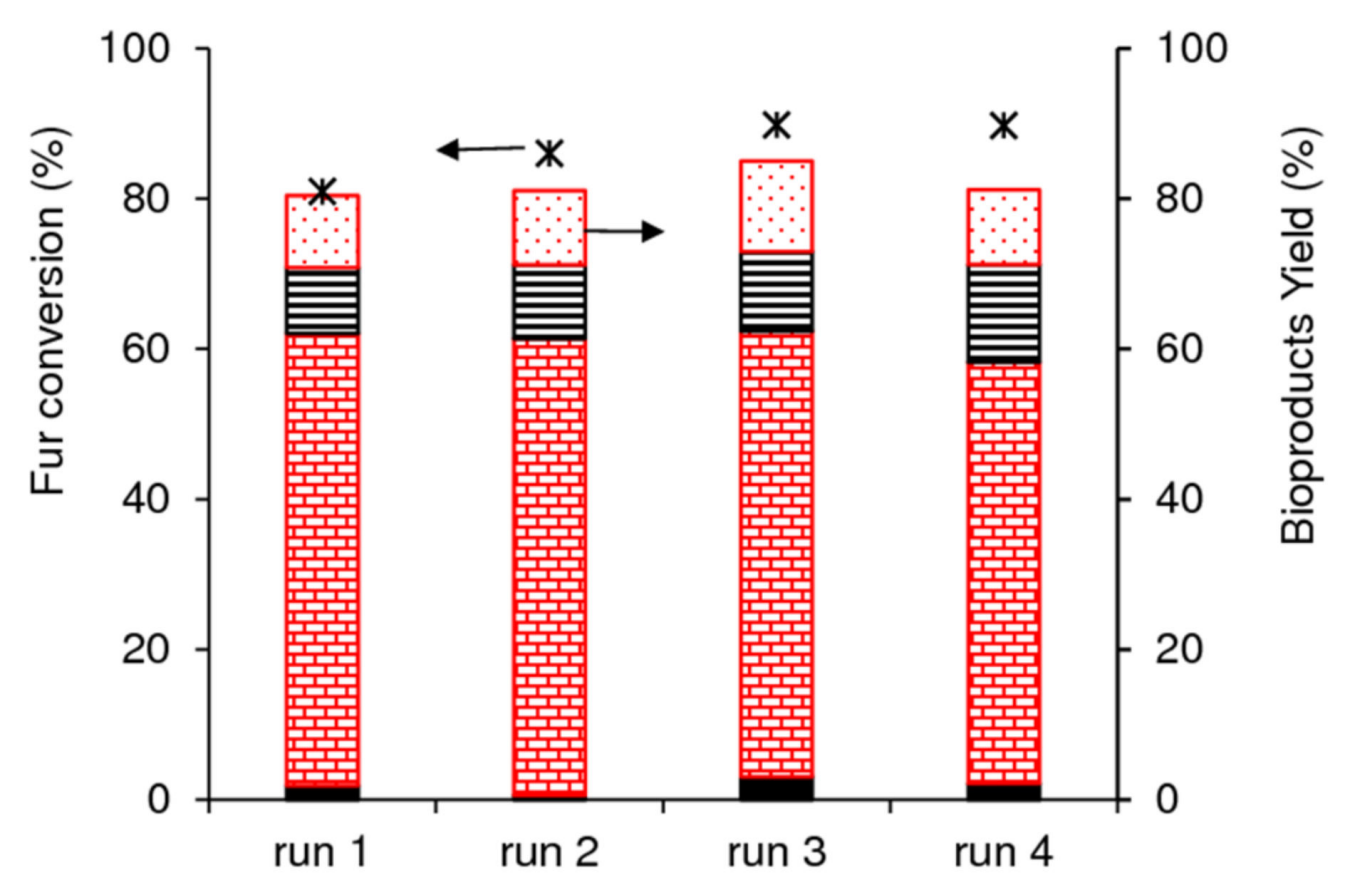
Consecutive batch runs of Fur reaction in the presence of Hf-TUD-1(75) at 150 °C; Fur conversion (*), and yield of FA (black bars), 2BMF (red bricks), 2BL (black stripes), AnL (red dots). Reaction conditions: 0.2 M Fur in 2BuOH, 20 g_cat_ L^−1^.

Hf-TUD-1(75), which led to higher furanic ether yields than the remaining catalysts, was used for four consecutive batch runs of Fur/2BuOH reaction at 150 °C/3 h, having performed steadily; Fur conversion and the bioproducts distributions remained similar (Figure 9). Hence, Hf-TUD-1(75) is a stable catalyst.

## 3. Materials and Methods

All reagents and solvents were obtained from commercial sources and were used as received. Please see the Supplementary Materials for details.

### 3.1. Synthesis of the Hf-TUD-1(x) Catalysts

Hf-containing TUD-1 materials were prepared via a one-pot sol–gel procedure under hydrothermal conditions, following a similar procedure to that described in Reference [93]. The materials prepared are denoted as Hf-TUD-1(x), where x stands for the molar ratio Si/Hf of the synthesis gel (x = 20, 50, 75). HfCl_4_ and TEOS were used as Hf and Si sources, respectively, and TEA and TEAOH were used as templating and mineralizing agents. The molar ratio composition of the gel was 1SiO_2_: yHfO_2_: 0.5TEAOH: 1TEA: 11H_2_O, where y = 0.040, 0.020, or 0.013 for x = 25, 50, and 75, respectively.

Specifically, HfCl_4_ was added to ca. 20 mL of 2PrOH, in an amount of 0.4 g (1.2 mmol), 0.4 g (1.2 mmol), or 0.067 g (0.2 mmol) for x = 25, 50, and 75, respectively. The resultant mixture was added slowly to TEOS (7.0 mL (30.8 mmol), 14.0 mL (61.5 mmol), and 3.5 mL (15.4 mmol) for x = 25, 50, and 75, respectively) and vigorously stirred for 10 min at room temperature. Then, a mixture of TEA (4.2 mL (30.8 mmol), 8.4 mL (61.5 mmol), and 2.1 mL (15.4 mmol) for x = 25, 50, and 75, respectively) and milli-Q water was added (6.1 mL (338 mmol), 12.2 mL (676 mmol), and 3.0 mL (169 mmol) for x = 25, 50, and 75, respectively). The initial molar ratio of TEA/H_2_O was 1:11 for all materials prepared. Subsequently, TEAOH was added (6.3 mL (15.4 mmol), 12.6 mL (30.8 mmol), and 3.2 mL (7.7 mmol) for x = 25, 50, and 75, respectively), and the mixture was vigorously stirred at room temperature for 2.5 h. The resultant mixture was aged under static conditions at room temperature for 24 h and then dried at 98 °C for 24 h. The obtained solid was gently grinded using an Agate mortar and pestle, followed by aging in a PTFE-lined stainless-steel autoclave at 178 °C for 24 h. The solids were subjected to Soxhlet extraction with ethanol for 4–6 h, dried overnight at 60 °C, and gently grinded using an Agate mortar and pestle. Finally, the solids were calcined at 600 °C under air flow for 10 h (heating rate of 1 °C min^−^^1^). For comparison, Zr-TUD-1(50) was prepared using a synthesis gel with Si/Zr = 50 (details in the Supplementary Materials).

### 3.2. Characterization of the Materials

The materials were characterized by complementary techniques. The details concerning the experimental parameters used for each technique are given in the Supplementary Materials (Characterization techniques). In summary, the structure, morphology, composition, and metal distributions were studied by X-ray powder diffraction (low- and wide-angle PXRD), scanning electron microscopy (SEM), energy dispersive X-ray spectroscopy (EDS), and elemental mappings at the micron-scale (SEM) and nanoscale (scanning transmission electron microscopy (STEM)). The textural parameters were calculated from the N_2_ adsorption isotherms measured at −196 °C: the specific surface area (S_BET_) was calculated using the Brunauer, Emmett, Teller equation; the total pore volume (V_p_) was calculated using the Gurvitch rule for relative pressure (p/p_0_) of at least 0.99; the mesopore size distribution curves were calculated by the DFT method (adsorption branch). The surface chemistry was characterized by Attenuated Total Reflectance (ATR) Fourier-transform Infrared FT-IR, ^29^Si magic-angle spinning (MAS), and cross polarization (CP) MAS nuclear magnetic resonance (NMR) spectroscopies. Elemental analyses for C of the used catalysts were obtained using a Leco TruSpec 630-200-200 analyser (Leco Instrumentos S.L, Madrid, Spain).

The acid properties were measured using a NexusThermo Nicolet apparatus (64 scans and resolution of 4 cm^−1^) equipped with a specially designed cell, using self-supported discs (5–10 mg cm^−2^) and pyridine as base probe. After in situ outgassing at 450 °C for 3 h under vacuum (10^–6^ mbar), pyridine (99.99%) was contacted with the sample at 150 °C for 10 min and subsequently evacuated at 150 °C or 350 °C for 30 min under vacuum. The measurements were based on the bands at ca. 1540 and 1455 cm^−1^, which are associated with pyridine adsorbed on Brønsted (B) and Lewis (L) acid sites, respectively (Figure S8) [54,55]. The strength of the B and L acid sites was based on the molar ratio B_350_/B_150_ and L_350_/L_150_, respectively, where B_150_ and B_350_ are the concentrations of B acid sites measured at 150 °C or 350 °C, respectively (likewise for L_150_ and L_350_).

### 3.3. Catalytic Tests

The catalytic reactions were carried out using tubular glass batch reactors with a conical bottom (partly homemade), equipped with a PTFE-coated magnetic stirring bar and a valve for purging. Each reactor was loaded with solvent, Fur (initial concentration in the range 0.2–2 M), and catalyst (5-35 g_cat_ L^−1^). The solvent (and reductant agent) was 2-butanol (2BuOH), 2-propanol (2-PrOH), 1-butanol (1BuOH), or ethanol (EtOH). Tests without catalyst or using HfO_2_, siliceous TUD-1, or Zr-TUD-1(50) as catalysts were performed for comparative studies.

The reactions of Hmf were carried out under the most favourable conditions encountered for the reaction of Fur (150 °C, 20 g_cat_ L^−1^, 0.2 M substrate, using 2BuOH and 2PrOH).

The charged reactors were immersed in a stirred, thermostatically controlled oil bath heated at the desired temperature (in the range 90–170 °C). The stirring rate of the reaction mixture was 1000 rpm to avoid diffusion limitations (Supplementary Materials, Influence of the stirring rate). The instant the reactors were immersed in the oil bath was considered the initial time. After a certain reaction time, the reactors were cooled to room temperature. Individual experiments were performed for a given reaction time, and the presented results are the mean values of at least two replicates (error < 7%).

Freshly prepared samples were analysed by high-performance liquid chromatography (HPLC) for quantification of the furanic aldehydes (Fur, Hmf) and by gas chromatography (GC) for quantification of the bioproducts. The HPLC analyses were carried out using a Knauer Smartline HPLC Pump 100 and a Shodex SH1011 H+ 300 mm × 8 mm (i.d.) ion exchange column (Showa Denko America, Inc., NY, USA) coupled to a Knauer Smartline UV (detector 2520; 254 nm; Paralab, Oporto, Portugal). The mobile phase was 0.005 M H_2_SO_4_(aq), 0.8 mL min^−1^, and column temperature of 50 °C. The GC analyses were carried out using an Agilent 7820A GC equipped with a capillary column (HP-5, 30 m × 0.320 mm × 0.25 mm) and a flame ionization detector.

Calibration curves with internal standards were measured for the quantification of the furanic aldehydes and bioproducts (please see Figure S17 for calibration curves exemplified for Hmf and Fur). The bioproducts were identified using a Shimadzu QP2010 ultra-GC-MS (Izasa Scientific, Lisbon, Portugal) equipped with a Zebron ZB-5MS capillary GC column (ZB-5, 30 m × 0.25 mm × 0.25 mm) and He as carrier gas (databases: Wiley229, NIST14). The bioproducts quantified for the Fur reaction system were furfuryl alcohol (FA), 2-(alkoxymethyl)furan ethers (AMFs; 2-(*sec*-butoxymethyl)furan (2BMF), 2-(isopropoxymethyl)furan (2PrMF), 2-(butoxymethyl)furan (BMF), and 2-(ethoxymethyl)furan (EMF)), levulinate esters (2-butyl levulinate (2BL), 2-propyl levulinate (2PrL), 1-butyl levulinate (BL), and ethyl levulinate (EL)), angelica lactone isomers (AnLs = α-angelica lactone β−angelica lactone), levulinic acid (LA), and the acetals 2-(diethoxymethyl)furan and 2-(dibutoxymethyl)furan (only formed in measurable amounts using EtOH and 1BuOH, respectively). For the HMF reaction system, 2,5-bis(alkoxymethyl) furans (BAMFs) were formed—namely, 2,5-bis(*sec*-butoxymethyl)furan (BBMF) using 2BuOH and 2,5-bis(isopropoxymethyl)furan (BPMF) using 2PrOH. The by-products were possibly non-volatile heavy products since only very small peaks sometimes appeared and could not be clearly identified.

The conversion (%) of the furanic aldehyde (Fal) at a reaction time *t* was calculated using the formula, 100 × [(initial molar concentration of Fal)−(molar concentration of Fal at reaction time t)/(initial molar concentration of Fal)], and bioproduct (Bpr) yield was calculated using the formula 100 × [(molar concentration of Bpr at time t)/(initial molar concentration of Fal)]. Initial reaction rates and initial activities were calculated based on Fal conversion at 1 h reaction. The carbon molar balance was calculated taking into consideration the amounts of total bioproducts formed and unconverted Fal.

The used catalysts were separated by centrifugation, washed using the same solvent as that used for the catalytic reaction, dried overnight at 85 °C, and finally treated at 600 °C (1 °C min^−1^) for 5 h under air flow (20 mLmin^−1^). The recovered catalysts were reused using the following conditions: 0.2 M Fur in 2BuOH, 150 °C, 20 g_cat_ L^−1^, 3 h. The contact test (CT) consisted in contacting the fresh catalyst with 2BuOH under the same conditions as those used for a normal catalytic test but without substrate (2BuOH at 150 °C, 20 g_cat_ L^−1^, 3 h); then the solid was separated by centrifugation (10,000 rpm), and the liquid phase was passed through a 220 nm pore size PTFE membrane; Fur was added to the obtained solution in an amount to give an initial concentration of 0.2 M; this solution was stirred for 3 h at 150 °C and finally analyzed by GC and HPLC.

### 3.4. Kinetic Modelling

Kinetic modelling studies were carried out for the more complex Fur reaction system. Scheme 2 shows a plausible overall reaction mechanism based on literature data [61,62]. To check whether this mechanism might apply to the catalytic systems of the present work, a kinetic model was developed (details in the Supplementary Materials) and model-fitting-based kinetic parameters were determined.

## 4. Conclusions

The conversion of furanic aldehydes—namely, furfural (Fur) and 5-(hydroxymethyl)furfural (Hmf)—to useful furanic ethers was effectively carried out using Hf-containing TUD-1 type mesoporous silicate catalysts (Hf-TUD-1(x), x = molar ratio Si/Hf) synthesized via a one-pot approach without using surfactants or expensive polymeric organic templates. The Hf sites played determinant catalytic roles in the reduction and acid chemistry involved in the conversion of Fur and Hmf to 2-(alkoxymethyl)furan ethers (AMFs) and bis(2-alkoxymethyl)furan (BAMFs), respectively. Enhanced acidity of Hf-TUD-1(x) (which were essentially Lewis acid catalysts with relatively few weak Brønsted acid sites), large mesopore sizes, and high pore volume seem favourable material properties for targeting AMF and BAMF.

The bioproducts spectrum was broader for the reaction of Fur than of Hmf. For the more complex Fur reaction system, a mechanism based on literature data (involving formation of furfuryl alcohol (FA), AMFs, levulinates esters, angelica lactones, and levulinic acid) was supported by kinetic modelling, which predicted that the fastest step was the conversion of FA to AMF. The Si/Hf ratio (x = 25, 50, 75) and reaction conditions (catalyst load, type of (secondary and primary) alcohol solvents and H-donor agents, reaction temperature (90–170 °C) and initial substrate concentration) were optimized to maximize the AMF yields; up to 65% 2-(*sec*-butoxymethyl)furan yield was reached at 84% conversion of Fur in 2-butanol at 4 h/150 °C, using Hf-TUD-1(75), which was a stable catalyst (based on reuse, contact tests, and characterization of the used solids).

The Hf-TUD-1(x) catalysts promoted the selective conversion of Hmf to BAMFs; e.g., 97% bis(2-butoxymethyl)furan selectivity at 96% Hmf conversion in 2-butanol at 21 h/150 °C, in the presence of Hf-TUD-1(75). This catalyst stood on a somewhat comparable or higher footing as other fully inorganic non-noble heterogeneous catalysts reported in the literature for Hmf conversion via CTH/alcohol strategies. To the best of our knowledge, this is the first study of hafnium-containing ordered mesoporous silicate catalysts used for the conversion of Fur or Hmf via CTH/alcohol strategies.

## Figures and Tables

**Table 1:** Composition, textural, and acid properties of the Hf-TUD-1(x) materials.

Sample	Si/Hf	S_BET_ (m^2^ g^−1^)	V_p_ (cm^3^ g^−1^)	D_p_ (nm)	Amount of Acid Sites ^1^ (µmol g^−1^)			L/B
					L	B	L + B	
Hf-TUD-75	67 ± 4	490	2.4	11–18	93	12	105	7.6
Hf-TUD-50	44 ± 5	660	2.0	6−10	120	10	130	12.0
Hf-TUD-25	23 ± 5	721	2.0	6–10	163	18	181	9.1

^1^ Measured by FT-IR spectroscopy of adsorbed pyridine (150 °C): L = Lewis acid sites; B = Brønsted acid sites.

**Table 2:** Best catalytic results for Hf-TUD-1(x) and comparison with literature data for Fur conversion to AMFs in alcohol medium over fully inorganic heterogeneous non-noble metal catalysts ^1^.

	Catalyst	T (°C)	t (h)	Solv.	[Fur]_0_	Cat/Fur (m/m)	C (%)	Y_AMF_ (%)	Reference
OMSi									
1	Hf-TUD-1(75)	150	4	2BuOH	0.2	1.0	88	63	-
2	Hf-TUD-1(50)	150	8	2BuOH	0.2	0.25	84	65	-
3	Zr-TUD-1(25)	120	7	2BuOH	0.45	0.6	70	55	[62]
4	Zr-MCM-41	120	3	2PrOH	1.31	0.21	44	20	[42]
5	HPMo(20)/Zr-MCM-41	150	24	2PrOH	0.05	8.33	76	20	[43]
6	Zr-SBA-15	130	6	2PrOH	0.27	1.0	100	86	[37]
7	Zr-SBA-15-RT	130	3	2PrOH	0.26	1.0	82	61	[36]
8	ZrO_2_/SBA-15, 17wt%Zr	150	2.5	2PrOH	0.26	0.4	100	70	[39]
Zeolitic materials									
9	ZrdeAl-Beta	120	3	2PrOH	1.31	0.21	50	41	[42]
10	ZrAl-Beta-n	150	7	2BuOH	0.45	0.32	95	35	[65]
11	ZrAl-Beta-n	120	7	2BuOH	0.45	0.6	85	55	[62]
12	MP-ZrAl-Beta-m	150	5	2BuOH	0.45	0.6	99	88	[65]
13	(Sn)_SSIE_-Beta	120	5	2BuOH	0.45	0.6	86	58	[61]
14	Zr-Beta-75-75	nm	0.33	2PeOH	0.2	0.52	24	17	[78]
15	Zr-HY-15-5	nm	0.33	2PeOH	0.2	0.52	50	36	[78]
16	2Hf-Al-USY-8	140	12	2PrOH	0.1	1.04	100	41	[79]
Metal oxides									
17	ZrO_2_	130	6	2PrOH	0.26	1.0	70	17	[80]
18	Co_3_O_4_-Al_2_O_3_	180	6	2PrOH	0.12	0.43	92	9	[81]
19	~70wt%ZrO_2_/SiO_2_	120	4	2BuOH	0.13	1.0	100	95	[34]
20	3.5wt%SiO_2_/ZrO_2_	140	1	2PrOH	0.07	0.42	56	49	[67]
21	16%Cu/SiO_2_	180	5	2PrOH	0.07	0.42	72	36	[67]
Others									
22	Zr-Mt+ZrO(OH)_2_^2^	100	2	2BuOH	0.4	0.8	100	69	[82]
23	APO-5	120	48	2PrOH	0.2	1.04	90	55	[83]
24	Zr-PO_4_	120	6	2PrOH	0.14	3.7	95	24	[84]
25	Zr-AlBeta/TUD-1	150	2	2BuOH	0.45	0.6	86	60	[65]
26	Ti3C_2_Tz MXene ^3^	180	48	2PrOH	0.3	50	62	13	[85]

^1^ Solv. = solvent, [Fur]_0_ = initial molar concentration of Fur, Cat = catalyst, C = conversion, Y_AMF_ = AMF yield, nm = not mentioned, PeOH = 2-pentanol. ^2^ Mt = montmorillonite. ^3^ 2D transition metal carbides and carbonitrides.

**Table 3:** Best catalytic results for Hf-TUD-1(x) and comparison with literature data for the conversion of Hmf to BBMF in alcohol medium over fully inorganic non-noble heterogeneous catalysts ^1^.

	Catalyst	T (°C)	t (h)	Solv.	[Hmf]_0_	Cat/Hmf (m/m)	C (%)	Y_BAMF_ (%)	Reference
OMSi									
1	Hf-TUD-1(75)	150	21	2BuOH	0.2	1	96	93	-
2	Hf-TUD-1(50)	150	3	2BuOH	0.2	1	80	79	-
3	Hf-TUD-1(50)	170	3	2BuOH	0.2	1	98	94	-
4	10%ZrO_2_/SBA-15	140	nm	2PrOH	0.08	0.1	12	8	[40]
5	10%ZrO_2_/SBA-15	180	nm	2PrOH	0.08	0.4	78	65	[40]
6	Zr-SBA-15	150	2.5	2PrOH	0.33	0.5	100	24	[41]
7	Zr-SBA-15	180	4	2PrOH	0.33	0.5	100	81	[41]
8	Zr-AlSBA-15	120	2.5	2PrOH	0.33	0.5	17	5	[41]
9	Zr-SBA-15-UH	150	3	2PrOH	0.06	0.5	96	91	[32]
10	Zr-SBA-15-UH	150	4	2BuOH	0.06	0.5	82	4	[32]
11	Zr-MCM-41	120	24	EtOH	0.06	0.03 ^2^	41	8	[44]
Zeolitic materials									
12	Sn-Beta	150	6	2PrOH	0.08	0.5	68	56	[33]
13	Sn-Beta	180	6	2PrOH	0.08	0.5	82	71	[33]
14	Sn-Beta	180	6	2BuOH	0.08	0.5	85	71	[33]
15	Hf-Beta	120	24	EtOH	0.06	0.03 ^2^	87	67	[44]
16	Zr-Beta	120	24	EtOH	0.06	0.03 ^2^	81	54	[44]
17	Sn-Beta	120	24	EtOH	0.06	0.03 ^2^	69	41	[44]
18	Hf-Beta	120	1	1BuOH	0.06	0.03 ^2^	84	22	[44]
19	Sn-Beta	140	nm	2PrOH	0.08	0.1	13	11	[40]
20	Sn-Beta	180	nm	2PrOH	0.08	0.4	70	61	[40]
21	Sn-SiBeta	95	8	2BuOH	0.27	0.32	15	15	[89]
22	Hf-Beta	120	1	2BuOH	0.06	0.03 ^2^	93	81	[44]
Others									
23	~70wt%ZrO_2_/SiO_2_	120	7	2BuOH	0.1	1	98	96	[34]
24	Zr-Mt+ZrO(OH)_2_ ^3^	150	1	2BuOH	0.4	0.8	100	96	[82]

^1^ Solv. = solvent, [Hmf]_0_ = initial molar concentration of Hmf, Cat = catalyst, C = conversion, Y_BAMF_ = yield of BAMF, nm = not mentioned. ^2^ Metal/Hmf molar ratio. ^3^ Mt = montmorillonite.

## Data Availability

The catalysts prepared are available from the authors upon request.

## Supplementary Materials

The following are available online. Details of Materials, synthesis of Zr-TUD-1(50) and characterization techniques, Figure S1: Influence of the stirring rates on Fur conversion, Table S1: Reaction kinetic constants (k_j_) of the modelled overall reaction of Fur, Figure S2: Kinetic model fitting to the experimental data for the reaction of Fur, Figure S3: SEM images of Hf-TUD-1(x), Figure S4: STEM images and element maps of Hf-TUD-1(x), Figure S5: Wide- and low-angle PXRD patterns of Hf-TUD-1(x), Figure S6: Wide- and low-angle PXRD patterns, N_2_ sorption isotherms, and mesopore size distribution of Zr-TUD-1(50), Figure S7: SEM images and element maps of Zr-TUD-1(50), Figure S8: FT-IR spectra of adsorbed pyridine, Figure S9: Influence of the reaction conditions on Fur conversion, Figure S10: Influence of the reaction conditions on bioproduct yields, Figure S11: Influence of the molar ratio Si/Hf of the Hf-TUD-1(x) catalysts, Figure S12: Influence of Hf-TUD-1(75) catalyst dosage, Figure S13: Influence of some reaction conditions on the reaction of Hmf int the presence of Hf-TUD-1(50), Figure S14: ATR FT-IR spectra of the fresh and used Hf-TUD-1(50), Figure S15: PXRD patterns of used Hf-TUD-1(x), Table S2: Composition, textural, and acid properties of used Hf-TUD-1(x), Figure S16: SEM images and corresponding element maps of used of Hf-TUD-1(x), Figure S17: Calibration curves.

## Sample Availability

Samples of the TUD-1 type materials herein reported are available from the authors.
