# Supplementary material for: Catalytic Transfer Hydrogenation and Acid Reactions of Furfural and 5-(Hydroxymethyl)furfural over Hf-TUD-1 Type Catalysts
Source: Molecules. 2021 Nov 27;26(23):7203. doi: 10.3390/molecules26237203 (PMC8658772; doi:10.3390/molecules26237203)
Supplement: Supplementary file 1 [file molecules-26-07203-s001.zip › molecules-1461695-supplementary.pdf]

SUPPLEMENTARY DATA

Catalytic Transfer Hydrogenation and Acid Reactions of Furfural and 5-(Hydroxymethyl)Furfural over Hf-TUD-1 Type Catalysts

CONTENTS

EXPERIMENTAL

Materials
Synthesis of Zr-TUD-1(50)
Characterization techniques
Influence of the stirring rate
Figure S1. Influence of the stirring rates on Fur conversion.

KINETIC MODELLING

Scheme S1. Plausible overall reaction mechanism for the reaction of Fur.
Table S1. Reaction rate constants (k_j) of the modelled overall reaction of Fur.
Figure S2. Kinetic model fitting to the experimental data for the reaction of Fur.

RESULTS OF CHARACTERIZATION AND CATALYTIC STUDIES

Characterization of the materials
Figure S3. SEM images of Hf-TUD-1(x).
Figure S4. STEM images and element maps of Hf-TUD-1(x).
Figure S5. Wide- and low-angle PXRD patterns of Hf-TUD-1(x).
Figure S6. Wide- and low-angle PXRD patterns, N_2 sorption isotherms, and mesopore size distribution of Zr-TUD-1(50) and TUD-1.
Figure S7. SEM images and element maps of Zr-TUD-1(50).
Figure S8. (a) FT-IR spectra of adsorbed pyridine at 150 ° of (a) Zr-TUD-1 (50), (b) Hf-TUD-1(25), (c) Hf-TUD-1(50) and (d) Hf-TUD-1(75).
Figure S9. Influence of the reaction temperature, type of solvent, initial Fur concentration and catalyst dosage on Fur conversion, in the presence of Hf-TUD-1(50).
Figure S10. Influence of the reaction temperature, type of solvent, initial Fur concentration and catalyst dosage on bioproduct yields for the reaction of Fur, in the presence of Hf-TUD-1(50).
Figure S11. Influence of the molar ratio Si/Hf of the Hf-TUD-1(x) catalysts.
Figure S12. Influence of the dosage of Hf-TUD-1(75) on the Fur reaction.
Figure S13. Influence of the type of solvent, reaction temperature and catalyst dosage on Hmf conversion and BAMF yields in the presence of Hf-TUD-1(50)
Figure S14. ATR FT-IR spectra of the fresh and used Hf-TUD-1(50).
Figure S15. PXRD patterns of used Hf-TUD-1(x).

Table S2. Composition, textural and acid properties of used Hf-TUD-1(x).
Figure S16. SEM images and corresponding element maps of used of Hf-TUD-1(x).
Figure S17. Calibration curves exemplified for Hmf and Fur.

EXPERIMENTAL

Materials

All reagents and solvents were obtained from commercial sources and used as received.

For materials syntheses: hafnium(IV) tetrachloride ($HfCl_4$; 98 %, Sigma-Aldrich, Algés, Portugal), zirconium(IV) tetrachloride ($ZrCl_4$; 99.5 %, Sigma-Aldrich, Algés, Portugal), tetraethylammonium hydroxide solution (TEAOH; 35 wt% in water, Aldrich, Algés, Portugal), tetraethylorthosilicate (TEOS; 98 %, Aldrich, Algés, Portugal), triethanolamine (TEA; 97 %, Fluka, Algés, Portugal), 2-propanol (99.5%, LabChem, Loures, Portugal), ethanol absolute anhydrous (EtOH; 99.9 %, Carlo Erba, Loures, Portugal).

For catalytic tests: furfural (99 %, Sigma-Aldrich, Algés, Portugal), 5-(hydroxymethyl)-furfural (Shangai, China), Hafnium oxide (excluding metals basis, 99.95%, Alfa Aesar, Kandel, Germany), 2-butanol (2BuOH, puriss, p.a., Reag. Ph. Eur., ≥ 99.5 %, Aldrich, Algés Portugal), 2-propanol (2PrOH, Honeywell, Riedel-de Häen™ Trace Select™, ≥ 99.9 % GC, Alfragide, Portugal), 1-butanol (1BuOH, Alfa aesar, > 99 %, Kandel, Germany), ethanol (EtOH, Honeywell, Riedel-de-Häen Trace Select™, ≥ 99.8 %, Alfragide, Portugal), Milli-Q water.

The siliceous TUD-1 sample was prepared as described in the main text for Hf-TUD-1(x) but without the Hf precursor; TUD-1 exhibited a type IV isotherm, S_{BET} = 592 m^2 g^{-1}, V_P = 1.88 cm^3 g^{-1}, median mesopore size of *ca.* 5 nm.

Synthesis of Zr-TUD-1(50)

0.072 g (0.31 mmol) $ZrCl_4$ was dissolved in *ca.* 8 mL of 2-propanol, at room temperature. This mixture was added slowly to 3.5 mL (15.4 mmol) TEOS, under vigorously stirring for 10 min. Then, a mixture of 2.1 mL (15.4 mmol) TEA and 3.0 mL (169 mmol) milli Q-water (molar ratio TEA/H_2O=1:11) was added, followed by the addition of 3.2 mL (7.7 mmol) TEAOH under vigorous stirring for 2.5 h. The resultant mixture was aged under static conditions at room temperature for 24 h and then dried at 98 °C for 24 h. The obtained solid was gently grinded using an Agate mortar and pestle, followed by aging in a PTFE-lined stainless-steel autoclave at 178 °C for 24. The solids were subjected to Soxhlet extraction with ethanol for 4-6 h, dried overnight at 60 °C, and gently grinded using an Agate mortar and pestle. Finally, the solid was calcined at 600 °C under air flow (20 mL min^{-1}) for 10 h (heating rate of 1 °C min^{-1}), giving Zr-TUD-1(50).

Characterization techniques

The X-ray powder diffraction (PXRD) data were collected on an Empyrean PANalytical diffractometer (Cu-Kα X-radiation, λ = 1.54060 Å) in a Bragg-Brentano *para*-focusing optics configuration (45 kV, 40 mA) at ambient temperature. Samples were prepared in a spinning flat plate sample holder and step-scanned in the range from 3° to 68° (2θ) with steps of 0.026°. A PIXEL linear detector with an active area of 1.7462° was used with a counting time of 68 s per step. Scanning electron microscopy (SEM) images, elemental mappings (Hf, Si, Al) and energy dispersive X-ray spectroscopy (EDS) analysis were obtained on a Hitachi SU-70 SEM microscope with a Bruker Quantax 400 detector operating at 20 kV. Scanning transmission electron microscopy (STEM) mapping images (Si and Hf) were collected using a Hitachi HD2700 scanning transmission electron microscope, equipped with a Bruker EDS detector; a drop of an ethanol suspension of solid sample was added to a holey amorphous carbon-film-coated 400 mesh copper grid (Agar Scientific). Nitrogen adsorption-desorption isotherms were measured at -196 °C, using a Quantachrome instrument (automated gas sorption data using Autosorb IQ$_2$). The samples were pre-treated at 250 °C for 3 h, under vacuum ($< 4 \times 10^{-3}$ bar). The specific surface area was calculated using the Brunauer, Emmett, Teller equation (S_{BET}) and the total pore volume (V_P) was based on the Gurvitch rule for relative pressure (p/p_0) of at least 0.99. The mesopore size distributions were determined by the DFT method (adsorption branch).

^{29}Si MAS NMR spectra were recorded at 79.495 MHz on a Bruker Avance III 400 MHz spectrometer (9.4 T), using a 7 mm double resonance MAS probe, with 2.4 μs radiofrequency pulse (40º flip angle pulse), a recycle delay of 60 s, and 5 kHz spinning

rate. Cross polarization (CP) $^{29}Si\{^1H\}$ CP MAS NMR spectra were measured with a 3.5 ms ^1H 90º flip angle pulse, 8000 ms contact time, recycle delay of 5 s and 5 kHz spinning rate. Chemical shifts are quoted in ppm from tetramethylsilane (TMS). Attenuated total reflectance (ATR) Fourier-transform infrared FT-IR spectra were recorded on a Unican Mattson Mod 7000 spectrometer equipped with a Specac Golden Gate Mk II ATR accessory fitted with a diamond top plate and KRS-5 focusing lenses (400-4000 cm^{-1}, 256 scans, 4 cm^{-1} resolution). Elemental analysis for C of the used catalysts were obtained using a Leco TruSpec 630-200-200 analyzer.

Influence of the stirring rate

Preliminary tests were carried out to check the effect of the stirring rate on the reaction kinetics (Hf-TUD-1-50, 0.2 M Fur in 2BuOH, 150 ºC) in order to avoid mass transfer limitations. Between 800 and 1200 rpm, the stirring rate did not influence considerably the reaction rate and the dependencies of the bioproducts yields on reaction time or on Fur conversion (Figure S1). The main difference was slightly higher maximum 2BMF yields reached using the stirring rate of 1000 rpm (57 % at 73 % Fur conversion). The catalytic tests were carried out at 1000 rpm.

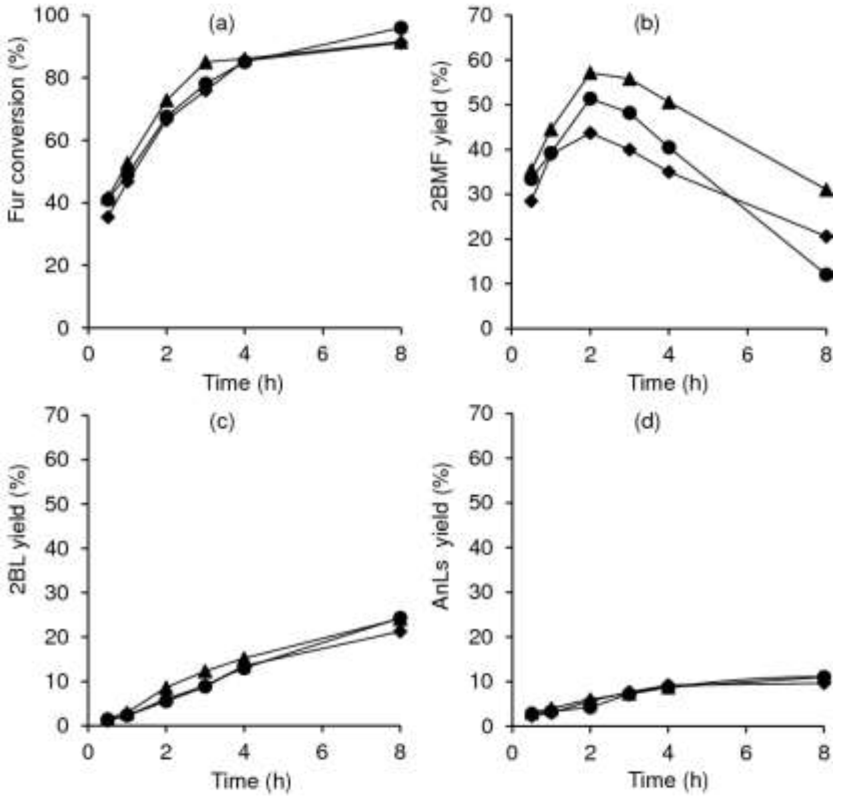

Figure S1. Influence of the stirring rate of 800 (◆), 1000 (▲) and 1200 rpm (●) on Fur conversion (a) and yields of 2BMF (b), 2BL (c) and AnLs (d), at 150 °C. Reaction conditions: 0.2 M Fur in 2BuOH, 20 g_{cat} L^{-1}, 150 °C.

KINETIC MODELLING

According to the literature, the conversion of Fur via CTH/alcohol strategies may proceed via an overall reaction mechanism presented in Scheme S1 [1-4]. Possible loss-reactions of the species involved were considered, since the carbon molar balances did not always close (discussed ahead); D_i denotes by-products formed from species i, where i = Fur, FA, 2BMF, AnLs, LA, 2BL.

The microreactors were modelled as perfectly stirred, isothermal batch reactors, and the mass balances are given by equation 1:

$$\frac{V}{W}\frac{\mathrm{d}C_{\mathrm{i}}}{\mathrm{d}t} = r_{\mathrm{i}} \tag{1}$$

where V is the reaction mixture volume (L), W is the mass of catalyst (g), C_i is the molar concentration of species *i* (M), t is time (h), and r_i is the overall reaction rate of species *i* expressed per unit of mass of catalyst (mol g_{cat}^{-1} h^{-1}); the ratio W/V was maintained constant in all experiments. The external diffusion limitations were neglected (please see the above section, *Influence of the stirring rate*). Internal diffusion limitations were neglected; all materials prepared where essentially characterized by mesopore sizes greater than 6 nm, which is much greater than the dimensions of the Fur molecules (< 0.6 nm length or width [5]).
A pseudo-homogeneous kinetic model was developed, considering first-order reactions according to equations 2-14:

$$\frac{V}{W}\frac{\mathrm{d}C_{\mathrm{Fur}}}{\mathrm{d}t} = -(k_1 + k_7)C_{\mathrm{Fur}} \tag{2}$$
$$\frac{V}{W}\frac{\mathrm{d}C_{\mathrm{FA}}}{\mathrm{d}t} = k_1 C_{\mathrm{Fur}} - (k_2 + k_8)C_{\mathrm{FA}} \tag{3}$$
$$\frac{V}{W}\frac{\mathrm{d}C_{\mathrm{2BMF}}}{\mathrm{d}t} = k_2 C_{\mathrm{FA}} - (k_3 + k_4 + k_9)C_{\mathrm{2BMF}} \tag{4}$$
$$\frac{V}{W}\frac{\mathrm{d}C_{\mathrm{AnLs}}}{\mathrm{d}t} = k_3 C_{\mathrm{2BMF}} - (k_5 + k_{10})C_{\mathrm{AnLs}} \tag{5}$$
$$\frac{V}{W}\frac{\mathrm{d}C_{\mathrm{LA}}}{\mathrm{d}t} = k_5 C_{\mathrm{AnLs}} - (k_6 + k_{11})C_{\mathrm{LA}} \tag{6}$$
$$\frac{V}{W}\frac{\mathrm{d}C_{\mathrm{2BL}}}{\mathrm{d}t} = k_4 C_{\mathrm{2BMF}} + k_6 C_{\mathrm{LA}} - k_{12} C_{\mathrm{2BL}} \tag{7}$$
$$\frac{V}{W}\frac{\mathrm{d}C_{\mathrm{D_{Fur}}}}{\mathrm{d}t} = k_7 C_{\mathrm{Fur}} \tag{9}$$
$$\frac{V}{W}\frac{\mathrm{d}C_{\mathrm{D_{FA}}}}{\mathrm{d}t} = k_8 C_{\mathrm{FA}} \tag{10}$$
$$\frac{V}{W}\frac{\mathrm{d}C_{\mathrm{D_{2BMF}}}}{\mathrm{d}t} = k_9 C_{\mathrm{2BMF}} \tag{11}$$
$$\frac{V}{W}\frac{\mathrm{d}C_{\mathrm{D_{AnLs}}}}{\mathrm{d}t} = k_{10} C_{\mathrm{AnLs}} \tag{12}$$
$$\frac{V}{W}\frac{\mathrm{d}C_{\mathrm{D_{LA}}}}{\mathrm{d}t} = k_{11} C_{\mathrm{LA}} \tag{13}$$
$$\frac{V}{W}\frac{\mathrm{d}C_{\mathrm{D_{2BL}}}}{\mathrm{d}t} = k_{12} C_{\mathrm{2BL}} \tag{14}$$

where k_j is the apparent reaction kinetic constant (L g_{cat}^{-1} h^{-1}) of step *j*. The kinetic constants were obtained using Matlab® software (version 9.5) by fitting the model (numerical integration with simultaneous optimization, using appropriate initial conditions, *i.e.* at t = 0) to the experimental data, minimizing the objective function (F_{obj}):

$$F_{obj} = \sum_{m}\left\{\sum_{n}\left(C_{m,n}\big|_{\mathrm{calc}} - C_{m,n}\big|_{\mathrm{exp}}\right)^2\right\} \tag{15}$$

where $C_{m,n}|_{\text{calc}}$ and $C_{m,n}|_{\text{exp}}$ are the concentrations predicted by the model and the experimental ones, respectively, for a specific specie m at each instant of time n.

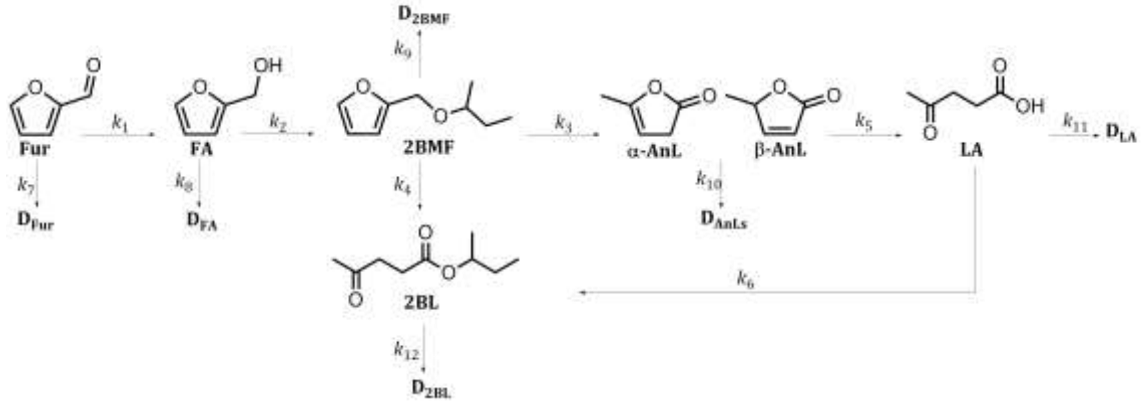

Scheme S1. Plausible overall reaction mechanism for the reaction of Fur over Hf-TUD-1-(x) type catalysts.

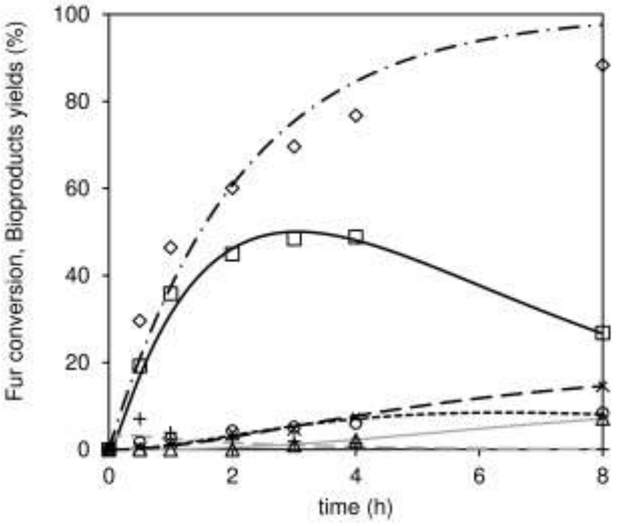

Figure S2. Dependency of the experimental data of Fur conversion (◊) on reaction time and the corresponding calculated kinetic curves (dash-point line). Dependency of the experimental yield of FA (+), 2BMF (□), 2BL (×), AnLs (○) and LA (Δ) on reaction time, and the calculated kinetic curves for FA (grey long-dash line), 2BMF (black solid line), 2BL (black long-dash line), AnLs (black short-dash), and LA (grey solid line). Reaction conditions: $[Fur]_0$ = 0.4 M in 2BuOH, catalyst load = 20 g_{cat} L^{-1}, 150 °C.

Table S1. Reaction kinetic constants (k_j) and average absolute relative deviation (AARD) of the modelled overall reaction of Fur, in the presence of Hf-TUD-50, using 2BuOH at 150 °C ($[Fur]_0$ = 0.4 M).[a]

Kinetic constant (L g_{cat}^{-1} h^{-1})	
k_1	2.24×10^{-2}
k_2	6.37×10^{-1}
k_3	3.63×10^{-3}
k_4	2.79×10^{-3}

k_5	9.96×10^{-3}
k_6	4.16×10^{-3}
k_7	1.03×10^{-5}
k_8	3.57×10^{-4}
k_9	5.59×10^{-3}
k_{10}	5.14×10^{-3}
k_{11}	0
k_{12}	3.13×10^{-3}
F_{obj}	0.0064
AARD (%)	18.6
$AARD_{FUR}$ (%)	3.3
$AARD_{FA}$ (%)	4.7
$AARD_{2BMF}$ (%)	0.7
$AARD_{AnLs}$ (%)	3.9
$AARD_{LA}$ (%)	2.6
$AARD_{2BL}$ (%)	3.3

[a]$AARD(\%) = \frac{100}{n_p}\sum_m\left\{\sum_n\left|\frac{C_{m,n}|_{\mathrm{calc}}-C_{m,n}|_{\mathrm{exp}}}{C_{m,n}|_{\mathrm{exp}}}\right|\right\}$, where $C_{m,n}\big|_{calc}$ and $C_{m,n}\big|_{exp}$ are the concentrations predicted by the model and the experimental ones, respectively, for a specific specie m at each instant of reaction time n, and n_p is the total number of experimental points. $AARD_i$ denotes the contribution of specie i to the overall AARD.

RESULTS OF CHARACTERIZATION AND CATALYTIC STUDIES

Characterization of the materials

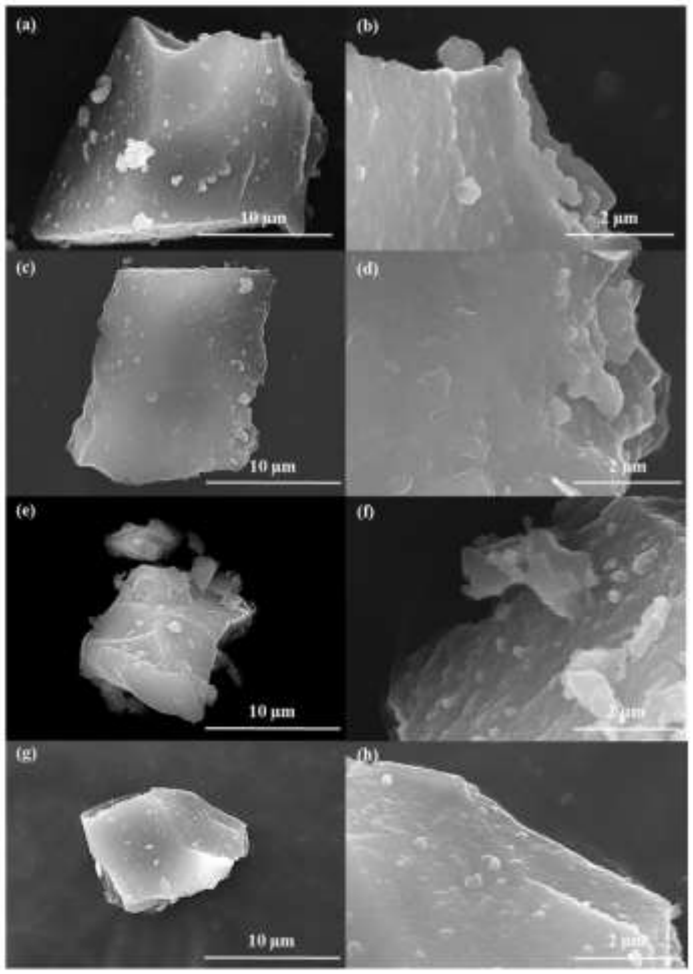

Figure S3. SEM images of Hf-TUD-1(75) (a,b), Hf-TUD-1(50) (c,d), Hf-TUD-1(25) (e,f) and TUD-1 (g,h).

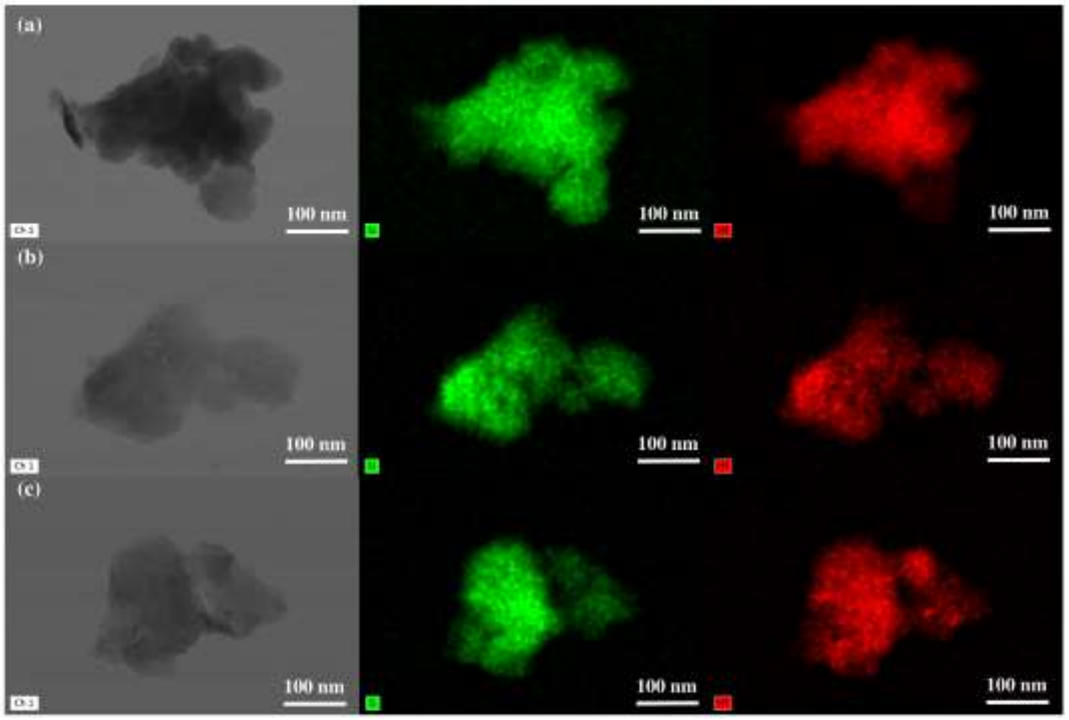

Figure S4. STEM images and corresponding elemental mappings (Si – green, Hf – red) of Hf-TUD-1(75) (a), Hf-TUD-1(50) (b) and Hf-TUD-1(25) (c).

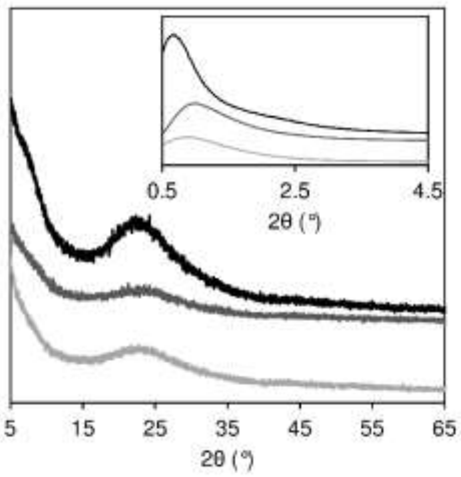

Figure S5. Wide-angle PXRD patterns of Hf-TUD-1(75) (black), Hf-TUD-1(50) (grey) and Hf-TUD-1(25) (light grey). Inset is the corresponding low-angle PXRD patterns (using the same colours).

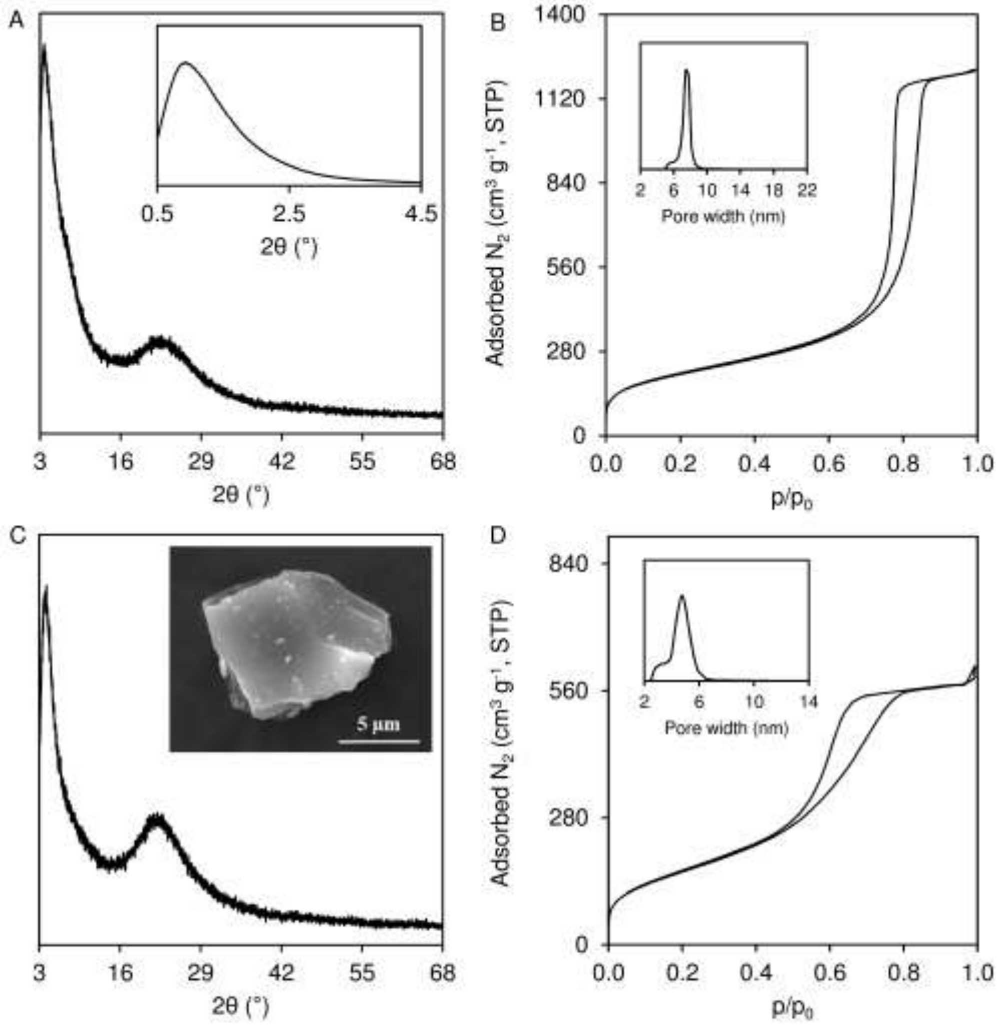

Figure S6. (a) Wide-angle PXRD pattern of Zr-TUD-1(50) (inset is the low-angle PXRD), and (b) N_2 sorption isotherm (at -196 °C) of Zr-TUD-1(50) (inset is the mesopore size distribution). (c) Wide-angle PXRD patter of TUD-1 (inset is the SEM image), and (d) N_2 sorption isotherm of TUD-1 (inset is the mesopore size distribution).

.

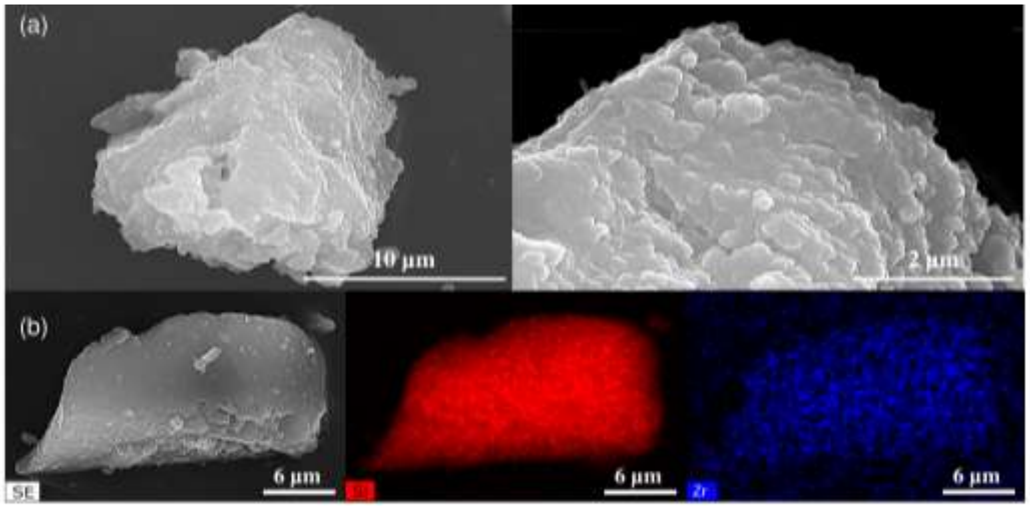

Figure S7. (a) SEM images and (b) elemental mappings (Si – red, Zr – blue) of Zr-TUD-1(50).

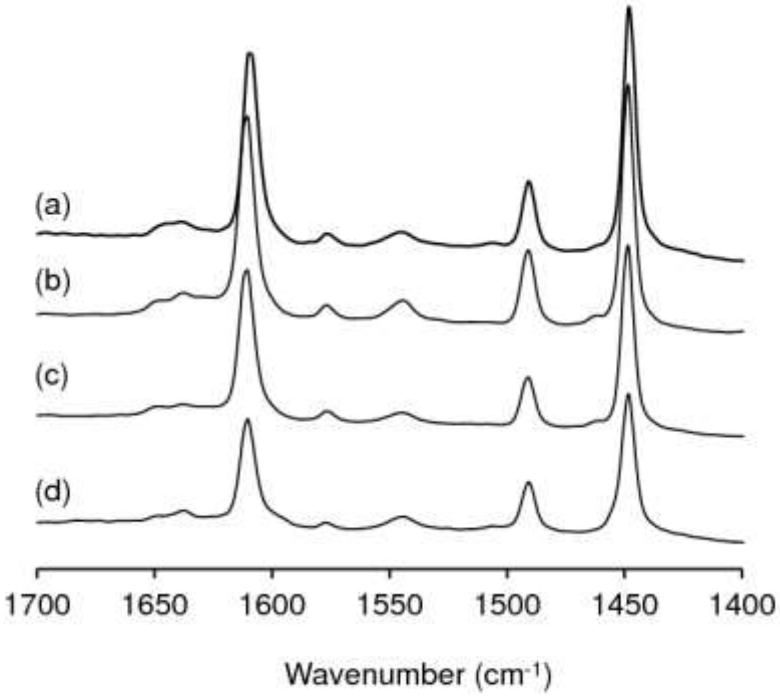

Figure S8. (a) FT-IR spectra of adsorbed pyridine at 150 °C of (a) Zr-TUD-1(50), (b) Hf-TUD-1(25), (c) Hf-TUD-1(50) and (d) Hf-TUD-1(75).

Catalytic studies

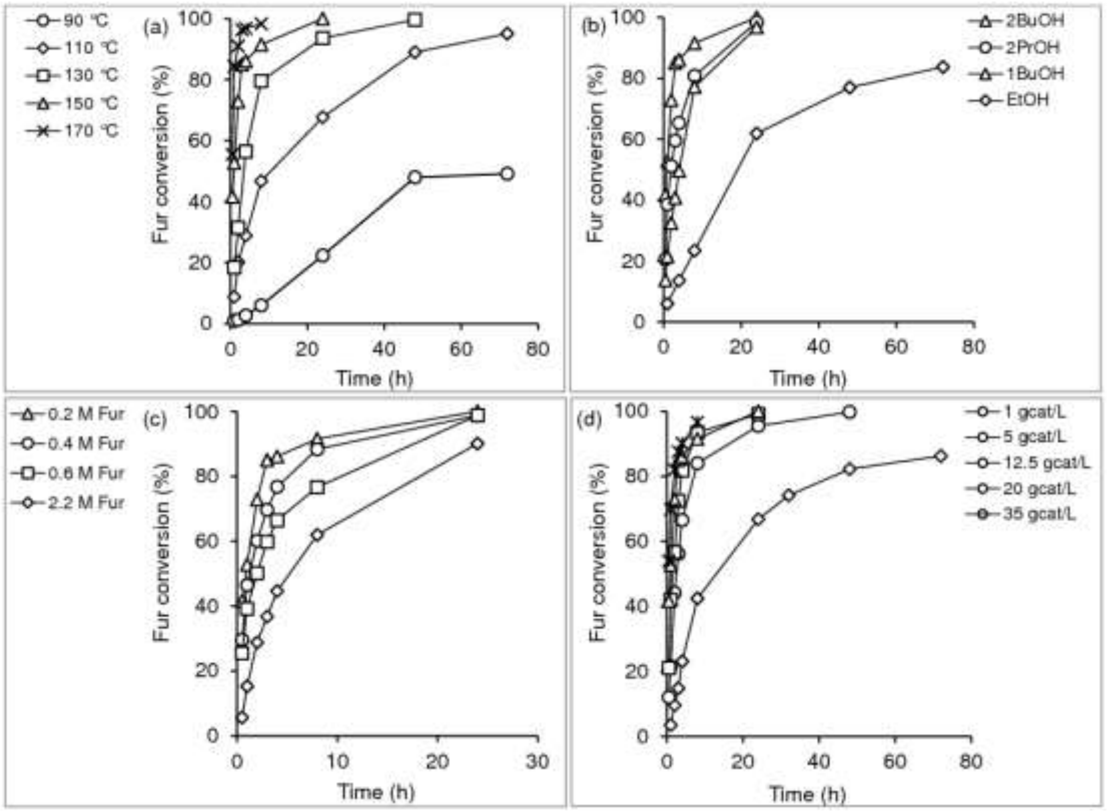

Figure S9. Influence of the reaction temperature (a), type of solvent (b), initial Fur concentration (c) and catalyst dosage (d) on the Fur conversion, in the presence of Hf-TUD-1(50). Reaction conditions: (a) 0.2 M Fur in 2BuOH, 20 g_{cat} L^{-1}; (b) 0.2 M Fur, 20 g_{cat} L^{-1}, 150 °C; (c) 2BuOH, 20 g_{cat} L^{-1}, 150 °C; (d) 0.2 M Fur in 2BuOH, 150 °C.

Figure S10. ***(Next page)*** - Influence of the reaction temperature (a-e), type of solvent (f-j), initial Fur concentration (k-o) and catalyst dosage (p-t) on bioproduct yields for the reaction of Fur, in the presence of Hf-TUD-1(50). Reaction conditions:
(a-e) 0.2 M Fur, 2BuOH, 20 g_{cat} L^{-1}; (f-j) 0.2 M Fur, 20 g_{cat} L^{-1}, 150 °C;
(k-o) 2BuOH, 20 g_{cat} L^{-1}, 150 °C; (p-t) 0.2 M Fur, 2BuOH, 150 °C.

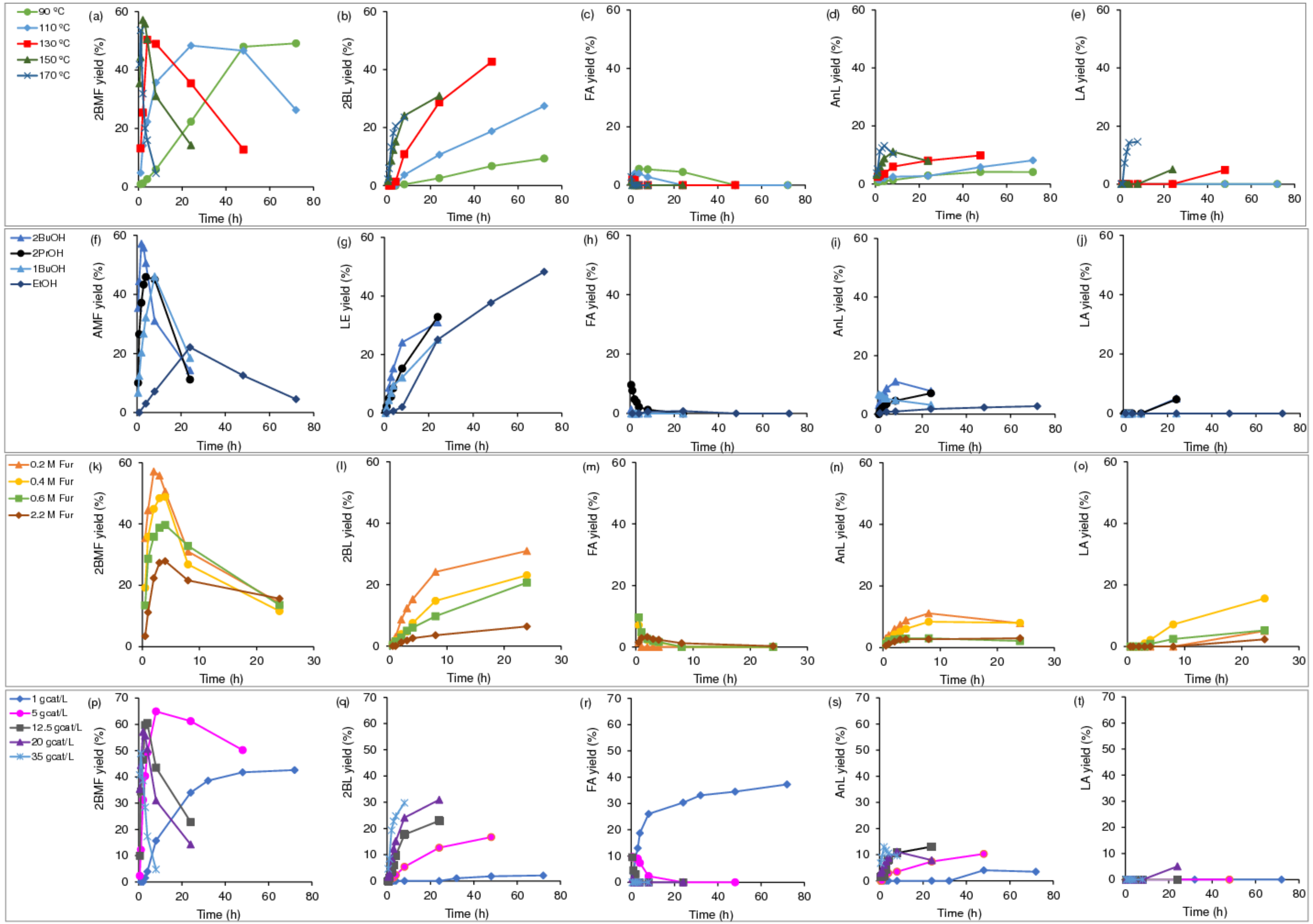

(a) 2BMF yield (%) Time (h)
90 °C
110 °C
130 °C
150 °C
170 °C
(b) 2BL yield (%) Time (h)
(c) FA yield (%) Time (h)
(d) AnL yield (%) Time (h)
(e) LA yield (%) Time (h)
(f) AMF yield (%) Time (h)
2BuOH
2PrOH
1BuOH
EtOH
(g) LE yield (%) Time (h)
(h) FA yield (%) Time (h)
(i) AnL yield (%) Time (h)
(j) LA yield (%) Time (h)
(k) 2BMF yield (%) Time (h)
0.2 M Fur
0.4 M Fur
0.6 M Fur
2.2 M Fur
(l) 2BL yield (%) Time (h)
(m) FA yield (%) Time (h)
(n) AnL yield (%) Time (h)
(o) LA yield (%) Time (h)
(p) 2BMF yield (%) Time (h)
1 gcat/L
5 gcat/L
12.5 gcat/L
20 gcat/L
35 gcat/L
(q) 2BL yield (%) Time (h)
(r) FA yield (%) Time (h)
(s) AnL yield (%) Time (h)
(t) LA yield (%) Time (h)

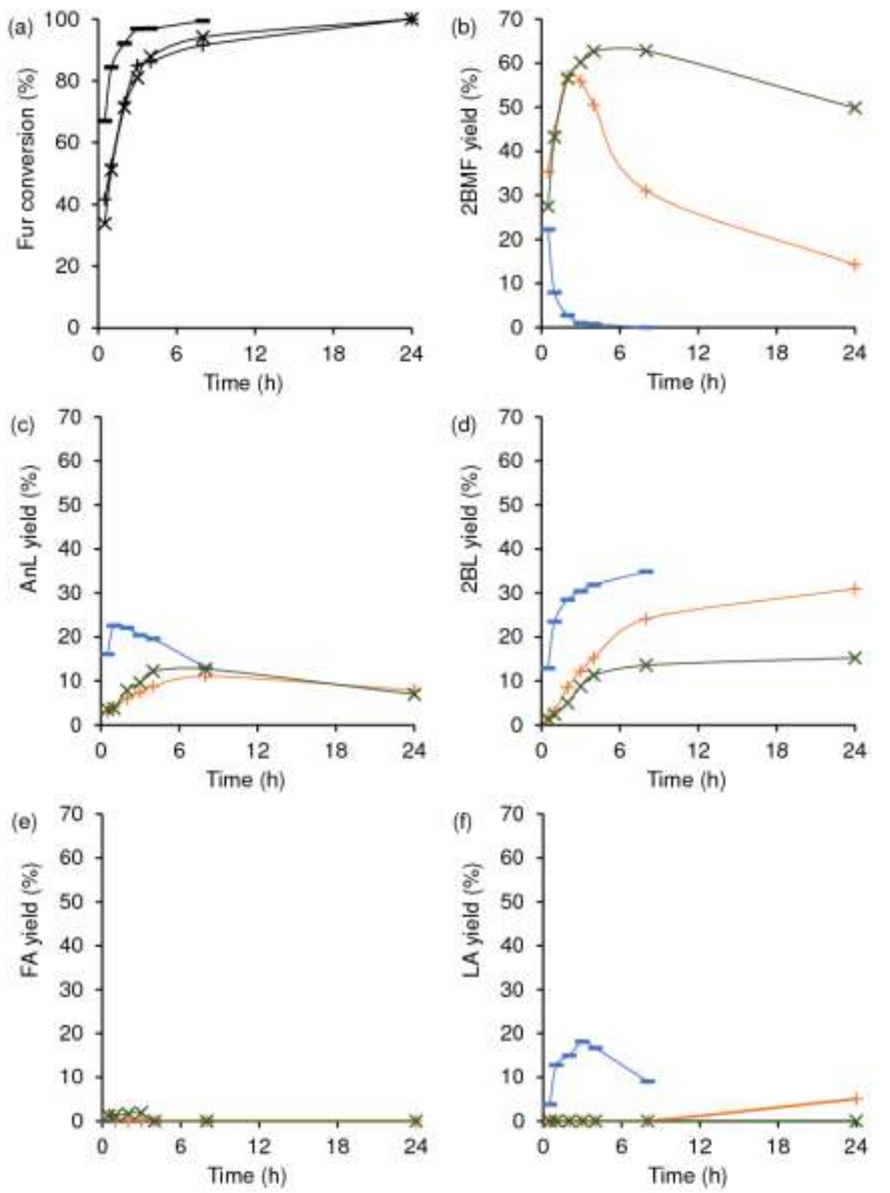

Figure S11. Influence of the molar ratio Si/Hf of the Hf-TUD-1(x) catalysts (x = 25 (-); x= 50 (+); x = 75 (×)) on Fur conversion (a) and yields of 2BMF (b), AnL (c), 2BL (d), FA (e) and LA (f) at 150 °C. Reaction conditions: 0.2 M Fur in 2BuOH, 20 g_{cat} L^{-1}, 150 °C.

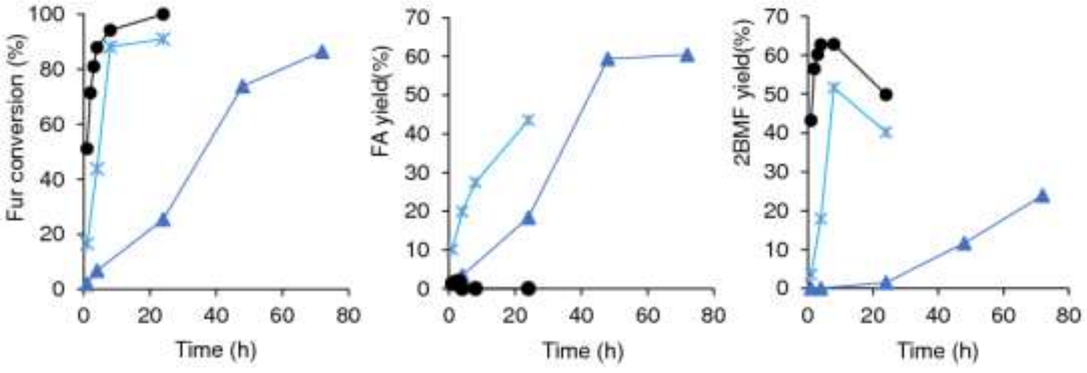

Figure S12. Influence of the dosage of Hf-TUD-1(75) on Fur conversion (a) and yields of FA (b) and 2BMF (c): 20 g_{cat} L^{-1} (●); 5 g_{cat} L^{-1} (*); 1 g_{cat} L^{-1} (●). Reaction conditions: 0.2 M Fur in 2BuOH, 150 °C.

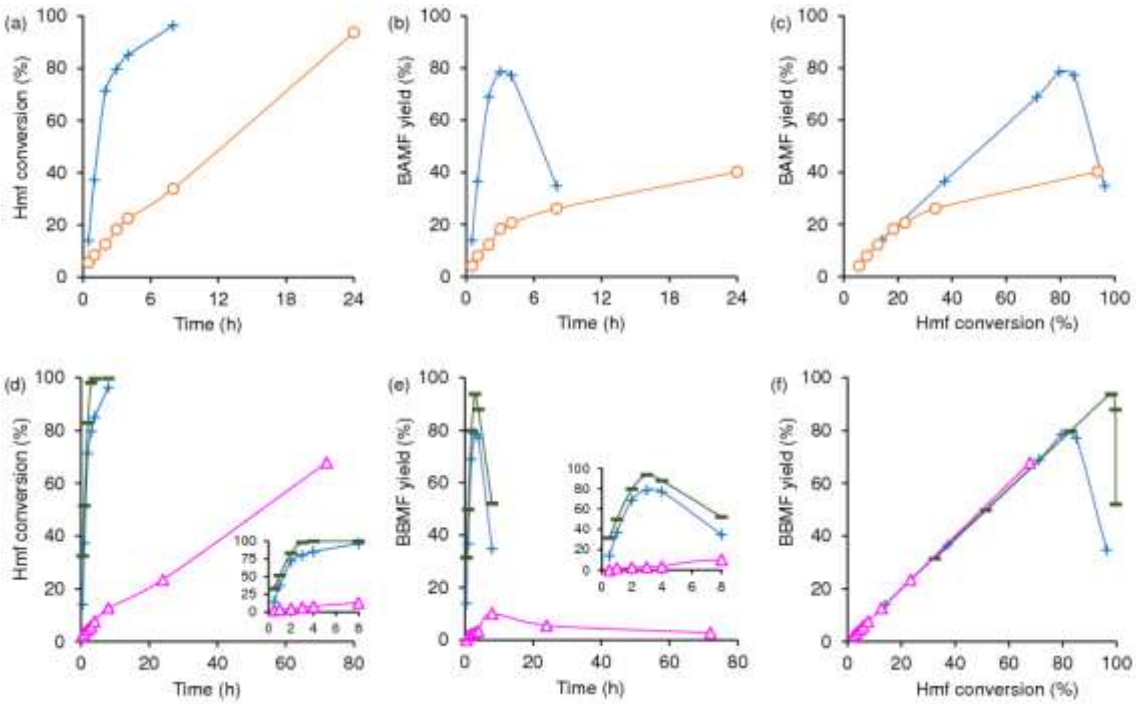

Figure S13. (a, b, c) Influence of the type of solvent (2BuOH (+); 2PrOH(o)) on Hmf conversion and BAMF yields, at 150 °C (0.2 M Hmf, 20 g_{cat} L^{-1}). (d, e, f) Influence of reaction temperature (150 °C (+); 170 °C (-) on Hmf conversion and BBMF yield (0.2 M Hmf, 20 g_{cat} L^{-1}), and of Hmf conversion to BBMF using a catalyst dosage of 1 g_{cat} L^{-1} at 150 °C (Δ) in the presence of Hf-TUD-1(50). The insets are given for a better visualization of the kinetic curves up to 8 h reaction.

Characterization of the used catalysts

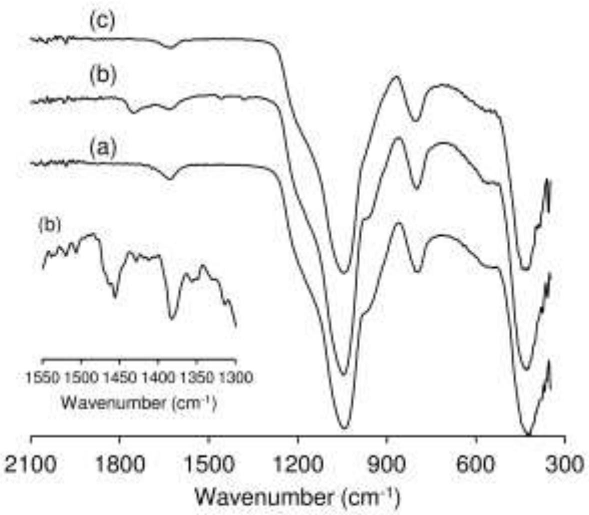

Figure S14. ATR FT-IR spectra of fresh Hf-TUD-1(50) (a), used/washed Hf-TUD-1(50) (b) and used/washed/calcined Hf-TUD-1(50) (c), after reaction with Fur. Reaction conditions: 0.20 M Fur, 1 mL 2BuOH, 150 ºC, 3 h.

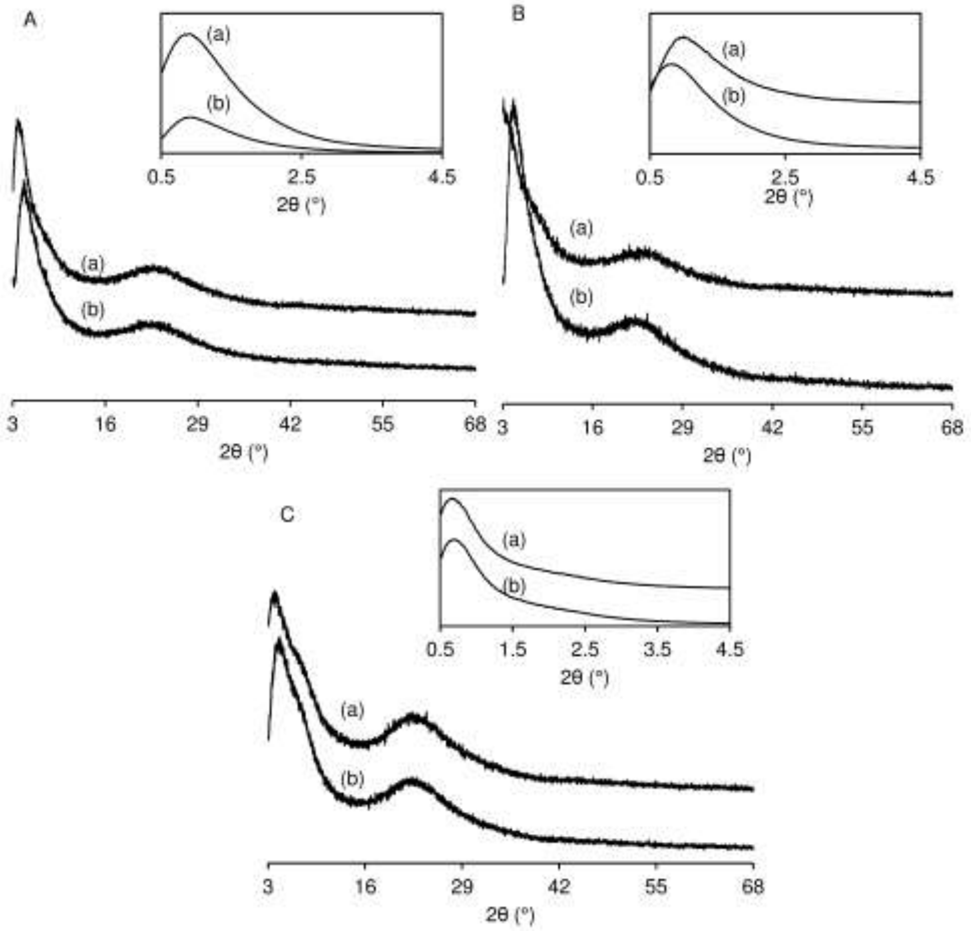

Figure S15. PXRD patterns of Hf-TUD-1(25) (A), Hf-TUD-1(50) (B) and Hf-TUD-1(75) (C) before (a) and after (b) use in the reaction of Fur, at 150 ºC. The insets are the corresponding low-angle PXRD patterns.

Table S2. Composition, textural and acid properties of the used catalysts (selected materials: Hf-TUD-1(x) with x = 50 and 75).

Sample	Si/Hf	S_{BET} (m^2 g^{-1})	V_p (cm^3 g^{-1})	D_p (nm)	Amount of acid sites (µmol g^{-1})			L/B
					L	B	L+B	
Hf-TUD-75	67	490	2.4	11-18	93	12	105	7.6
Hf-TUD-75-used	65	479	2.3	12-19	87	8	95	11.6
Hf-TUD-50	44	660	2.0	6-10	120	10	130	12.0
Hf-TUD-50-used	39	646	2.0	6-10	125	10	135	12.5

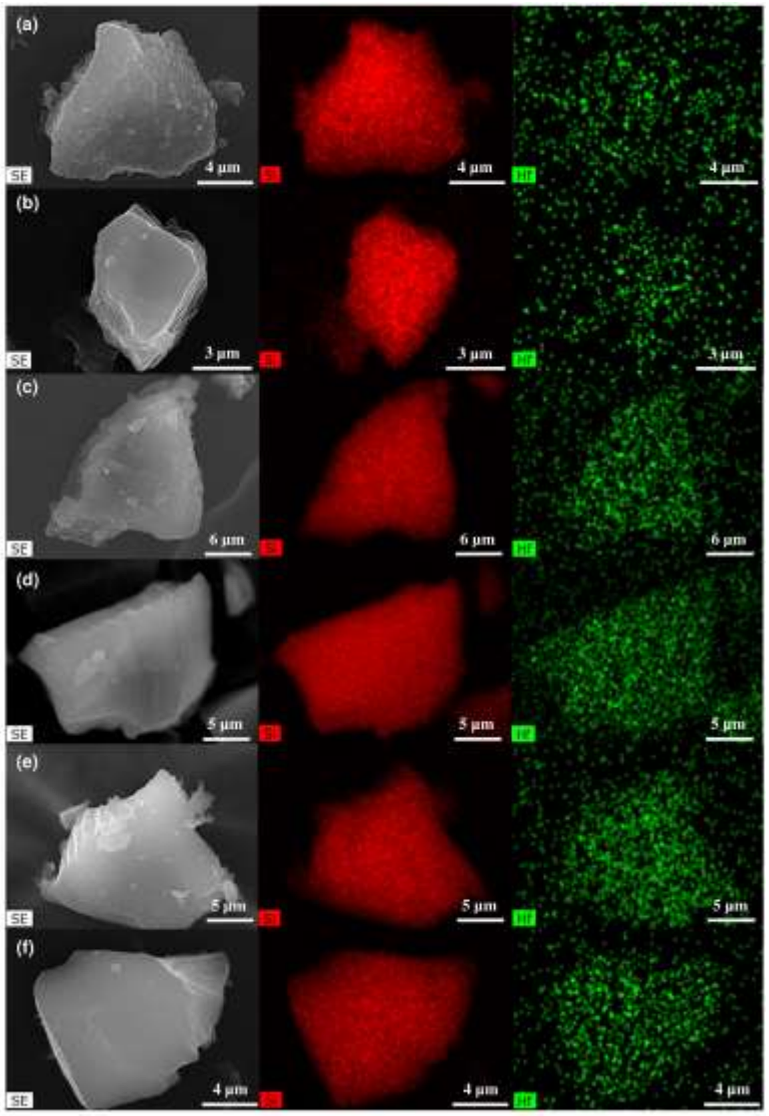

Figure S16. SEM images and corresponding element maps (Si – red, Hf – green) of Hf-TUD-1(75) (fresh (a), used (b)), Hf-TUD-1(50) (fresh (c), used (d)), and Hf-

TUD-1(25) (fresh (e), used (f)). Reaction conditions: 0.2 M Fur in 2BuOH, 20 g_{cat} L^{-1}, 150 ºC.

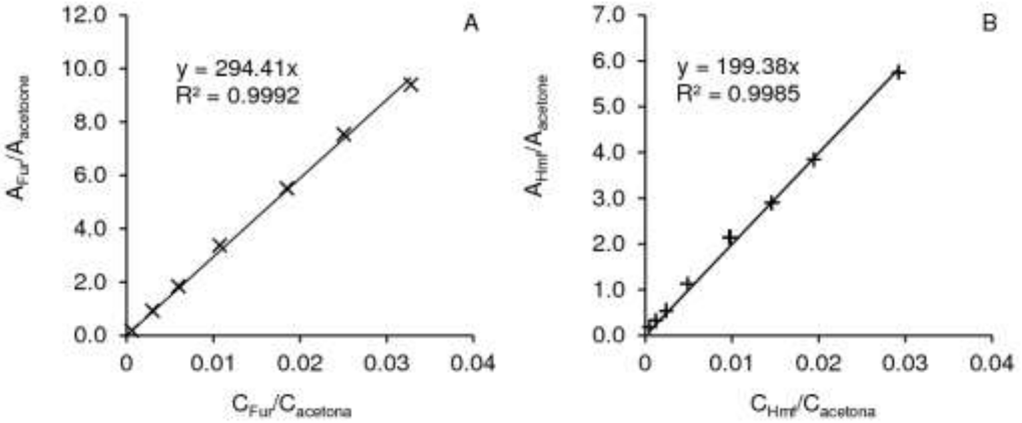

Figure S17. Chromatography calibration curves exemplified for Hmf and Fur.

REFERENCES

[1] M. M. Antunes, S. Lima, P. Neves, A. L. Magalhães, E. Fazio, A. Fernandes, F. Neri, C. M. Silva, S. M. Rocha, M. F. Ribeiro, M. Pillinger, A. Urakawa, A. A. Valente, One-Pot Conversion of Furfural to Useful Bioproducts in the Presence of a Sn, Al-Containing Zeolite Beta Catalyst Prepared via Post-Synthesis Routes, *J. Catal.* **2015**, *329*, 522-537.

[2] M. M. Antunes, S. Lima, P. Neves, A. L. Magalhães, E. Fazio, F. Neri, M. T. Pereira, A. F. Silva, C. M. Silva, S. M. Rocha, M. Pillinger, A. Urakawa, A. A. Valente, Intgrated reduction and Acid-Catalysed Conversion of Furfural in Alcohol Medium Using Zr, Al-Containing Ordered Micro/Mesoporous Silciates, *Appl. Catal. B: Environ*. **2016**, *182*, 485-503.

[3] H. P. Winoto, B. S. Ahn, J. Jae, Production of g-Valerolactone from Furfural by a Single Step Process Using Sn-Al-Beta Zeolites: Optimizing the Catalyst Acid Properties and Process Conditions, *J. Ind. Eng. Chem*. **2016**, *40*, 62-71.

[4] H. P. Winoto, Z.A. Fikri, J.-M. Ha, Y.-K. Park, H. Lee, D.J. Suh, J. Jae, Heteropolyacid Supported on Zr-Beta Zeolite as an Active Catalyst for One-Pot Transformation of Furfural to g-Valerolactone, *Appl. Catal. B: Environ.* **2019**, *241*, 588-597.

[5] J. Jae, G.A. Tompsett, A.J. Foster, K.D. Hammond, S.M. Auerbach, R.F. Lobo, G.W. Huber, Investigation into the Shape Selectivity of Zeolite Catalysts for Biomass Conversion, *J. Catal*. **2011**, *279* 257–268.
